# A longitudinal prospective study of active tuberculosis in a Western Europe setting: insights and findings

**DOI:** 10.1007/s15010-024-02184-2

**Published:** 2024-02-13

**Authors:** Arantxa Romero-Tamarit, Xavier Vallès, María Munar-García, Juan Espinosa-Pereiro, Núria Saborit, Ma. Teresa Tortola, Zoran Stojanovic, Sílvia Roure, Adrián Antuori, Pere-Joan Cardona, Antoni Soriano-Arandes, Andrea Martin-Nalda, María Espiau, Maria Luiza de Souza-Galvão, Ma. Ángeles Jiménez, Antoni Noguera-Julian, Israel Molina, Xavier Casas, Marisol Domínguez-Álvarez, Neus Jové, Nino Gogichadze, Kaori L. Fonseca, Lilibeth Arias, Joan-Pau Millet, Adrián Sánchez-Montalvá, Cristina Vilaplana

**Affiliations:** 1 Unitat de Tuberculosi Experimental, Germans Trias i Pujol Research Institute (IGTP), Can Ruti Campus, Ctra. del Canyet, S/N, 08916 Badalona, Spain; 2 Autonomous University of Barcelona, Bellaterra, Spain; 3 North Metropolitan International Health Program (PROSICS), Badalona, Spain; 4 Territorial Clinical Directorate on Infectious Diseases and International Health Clinical Division within the Northern Metropolitan Management of the Catalan Institute of Health, Badalona, Spain; 5 Fundació Lluita Contra les Infeccions, Hospital Germans Trias i Pujol, Badalona, Spain; 6 Pompeu Fabra University, Barcelona, Spain; 7 Infectious Diseases Department, Vall d’Hebrón University Hospital, Universitat Autónoma de Barcelona, Barcelona, Spain; 8 International Health Program of the Catalan Institute of Health (PROSICS), Barcelona, Spain; 9 CIBER of Infectious Disease (CIBERINFEC), Instituto de Salud Carlos III, Madrid, Spain; 10 Microbiology Department, Hospital Vall d’Hebron, Barcelona, Spain; 11 Mycobacterial Infection Study Group from the Spanish Society of Clinical Microbiology and Infectious Diseases (GEIM-SEIMC), Barcelona, Spain; 12 Pneumology Department, Hospital Universitari Germans Trias I Pujol, Badalona, Spain; 13 CIBER Respiratory Diseases (CIBERES), Instituto de Salud Carlos III, 28029 Madrid, Spain; 14 Infectious Diseases Department, Germans Trias i Pujol Hospital and Research Institute, 08916 Badalona, Spain; 15 Microbiology Department, Northern Metropolitan Clinical Laboratory, Hospital Universitari Germans Trias I Pujol, Badalona, Spain; 16 Paediatric Infectious Diseases and Immunodeficiencies Unit, Hospital Vall d’Hebron, Barcelona, Spain; 17 Malalties Infeccioses i Resposta Inflamatòria Sistèmica en Pediatria, Servei de Malalties Infeccioses i Patologia Importada, Institut de Recerca Pediàtrica Sant Joan de Déu, 08950 Barcelona, Spain; 18 CIBER of Epidemiology and Public Health (CIBERESP), Instituto de Salud Carlos III, 28029 Madrid, Spain; 19 Departament de Cirurgia i Especialitats Medicoquirúrgiques, Facultat de Medicina i Ciències de la Salut, Universitat de Barcelona, 08036 Barcelona, Spain; 20 Red de Investigación Traslacional en Infectología Pediátrica RITIP, 28029 Madrid, Spain; 21 Serveis Clínics de Barcelona, Barcelona, Spain; 22 Hospital del Mar, Barcelona, Spain; 23 Epidemiology Service, Barcelona Public Health Agency, Barcelona, Spain

**Keywords:** *Mycobacterium tuberculosis*, Inflammatory parameters, HRQoL, Clinical monitoring, Biomarkers

## Abstract

**Purpose:**

This study investigates the potential of inflammatory parameters (IP), symptoms, and patient-related outcome measurements as biomarkers of severity and their ability to predict tuberculosis (TB) evolution.

**Methods:**

People with TB were included prospectively in the Stage-TB study conducted at five clinical sites in Barcelona (Spain) between April 2018 and December 2021. Data on demographics, epidemiology, clinical features, microbiology, and Sanit George Respiratory Questionnaire (SGRQ) and Kessler-10 as Health-Related Quality of Life (HRQoL) were collected at three time points during treatment. C-reactive protein (CRP), erythrocyte sedimentation rate (ESR), neutrophil/lymphocyte, and monocyte/lymphocyte ratios (NLR and MLR), complement factors C3, C4, and cH50, clinical and microbiological data, and HRQoL questionnaires were assessed at baseline, 2 months, and 6 months. Their ability to predict sputum culture conversion (SCC) and symptom presence after 2 months of treatment was also analysed.

**Results:**

The study included 81 adults and 13 children with TB. The CRP, ESR, NLR, and MLR values, as well as the presence of symptoms, decreased significantly over time in both groups. Higher IP levels at baseline were associated with greater bacillary load and persistent symptoms. Clinical severity at baseline predicted a delayed SCC. Kessler-10 improved during follow-up, but self-reported lung impairment (SGRQ) persisted in all individuals after 6 months.

**Conclusions:**

IP levels may indicate disease severity, and sustained high levels are linked to lower treatment efficacy. Baseline clinical severity is the best predictor of SCC. Implementing health strategies to evaluate lung function and mental health throughout the disease process may be crucial for individuals with TB.

**Supplementary Information:**

The online version contains supplementary material available at 10.1007/s15010-024-02184-2.

## Introduction

Active tuberculosis (TB) diagnosis involves observing symptoms, conducting radiographic imaging, and confirming with microbiological tests [1]. Treatment monitoring is based on microbiological status combined with clinical and radiological evaluation [2]. Moreover, the management of TB in children presents differences and additional difficulties; as the disease tends to be paucibacillary, there is a lack of specific paediatric diagnostic tests and diagnosis often relies on clinical assessment [3, 4]. The lack of satisfactory tools for monitoring the efficacy of TB therapy prevents a personalised management of people with TB. However, new approaches have been put forward in the last few years [5–7]. The management of TB control tends to prioritize the microbiological-based cure [8]. While this is crucial from a public health standpoint, it fails to sufficiently address the physical, mental, and social impact of TB [9].

Mediators driving inflammation play a critical role in active TB pathogenesis and recent studies highlight the intimate relationship between inflammatory biomarkers as C-Reactive Protein (CRP) and disease severity [10, 11]. Erythrocyte Sedimentation Rate (ESR) serves as a non-specific indicator fluctuating during infectious processes, signaling inflammation, and treatment response magnitude in TB [12]. During Mtb infection, blood cell production undergoes alterations, impacting neutrophil–lymphocyte ratios (NLR) and monocyte–lymphocyte ratios (MLR). These ratios reflect immune responses and may serve as cost-effective, readily available biomarkers for TB recurrence and survival, with altered MLR potentially preceding active TB, indicating severe disease and sputum conversion delays in intensive treatment phases [10, 13].

Furthermore, the perceptions of individuals about their disease and health status are poorly documented, and there is no standardised tool to do this [14, 15]. Patient-reported quality-of-life (QoL) tools, such as the Saint George Respiratory Questionnaire (SGRQ) [16] and Kessler-10 [17], could help us to understand the impact of TB on people and, therefore, provide them with extra support to overcome this disease.

This study aimed to prospectively follow up a cohort of TB people in the Barcelona area, to investigate the potential of inflammatory parameters (IP), symptoms, and patient-related outcome measures as biomarkers of severity, and their ability to predict the evolution of the people with TB.

## Methods

### Study design and population

We report the results of the participants enrolled in the STAGE-TB cohort between April 2018 and December 2021. The STAGE-TB (NCT03691883) is a longitudinal prospective cohort conducted at five major healthcare centers acting as clinical sites in Barcelona (Spain), and is actively enrolling participants. Initially, a sample size of *n* = 200 individuals with TB was projected to be enrolled over a span of approximately 2 years. This estimation was rooted in the 2017 count of diagnosed TB cases within the included centers at the project's inception.

The inclusion criteria for the STAGE-TB are: probable or confirmed TB and agreement to participate; and consenting to data/sample donation. Patients who did not consent to data/sample donation were excluded. We included consecutive adults and children with no age restriction diagnosed with pulmonary and extrapulmonary tuberculosis at, or referred to, any of the sites.

### Data and sample collection

Data were recorded at baseline (BL; within the first 2 weeks of TB diagnosis), at month 2 and 6 after treatment initiation (FUM2 and FUM6). For those requiring treatment or clinical follow-up beyond 6 months (i.e. those suffering of DR-TB), extra-visits for sampling and variables collection were scheduled every 6 months and at the final visit. However, for the present study, we have only analysed the data up to FUM6 for all individuals enrolled. The data were entered into an electronic case report form (eCRF) created ad hoc on the REDCap platform (www.project-redcap.org).

At each time point, we collected clinical (medical history, symptoms), microbiological (sputum smear and culture results), and IP data. CRP (mg/L), ESR (mm/h), NLR, MLR, complement CH50 (mg/dL), C3 (mg/dL), and C4 (mg/dL) levels were selected for their affordability and widespread availability in clinical laboratories; and measured at each site’s laboratory using commercial kits.

In adults, the impact on overall health, daily life, and perceived well-being was assessed. The impact on lung function was evaluated in individuals with pulmonary TB using the SGRQ, a standardized self-administered survey created to assess perceived well-being in individuals with airways diseases [16], consisting of 76 items divided into 3 domains: (a) symptoms, (b) activity, and (c) psychosocial impact. The value ranges from 0 (no reduction in quality of life) to 100 (maximum reduction in quality of life) [16]. We analysed the SGRQ total score, which normal values are between 5 and 7 points and incorporates scores from each component of the SGRQ.

The psychological distress was measured using the Kessler-10 questionnaire, an instrument that comprises ten specific questions addressing psychological distress [17], specifically symptoms of anxiety and depression experienced in the preceding 4 weeks. Respondents use Likert-type scales with five levels: always, almost always, sometimes, almost never, and never. A scalar value of 1 is assigned to the response "never," while "always" is assigned a value of 5 points. The total score ranges from 10 to 50, reflecting the sum of individual question scores [17].

The outcome variables as surrogates of improvement were defined as culture conversion time (fast converters: ≤ FUM2; slow converters: > FUM2), and presence/absence of symptoms at FUM2.

### Statistical analysis

The median and interquartile range (IQR) or frequency and percentage were calculated, as appropriate. Normality was not assumed. Fisher’s test and the Wilcoxon–Mann–Whitney test were used to compare categorical and continuous variables, respectively, with paired-data corrections if necessary. The evolution of each parameter during follow-up visits was stratified based on relevant demographic, clinical, and epidemiological factors. Logistic regression was used to predict the outcomes of interest (culture conversion and presence of symptoms at month 2), adjusting each biomarker by age. Collinearity between parameters analysed was excluded through a variance analysis. CRP and ESR were dichotomized using the thresholds applied in clinical practice (5 mg/L and 20 mm/h, respectively). NLR, MLR, and complements were categorized as being over or under their median BL value, and the clinical severity as having up to three symptoms *vs.* four symptoms or more. The data from adults and children were analysed separately. The SGRQ and Kessler-10 analysis included those adults who at least had the questionnaire completed at baseline. The receiver operator curve (ROC) was constructed and cut-off value using the Youden index [18] for each IP, and the performance values of the selected cut-off values expressed in terms of sensitivity and specificity.

All analyses were performed with Stata statistical software version 12.0. A significance level of 5% was considered for all tests.

## Results

### Characteristics of the cohort

A total of 94 participants were included in the study, with 27.6% being female and 13.8% under the age of 18 years. The median age and IQR of the participants was 43 years (27–55). In total, more than half of cases were pulmonary TB, and the median diagnostic delay was 45 days. Of the total, 47.9% were of Spanish origin. Among the adults, 34.4% were active smokers and 16.8% reported daily or almost daily alcohol intake. Diabetes was the most frequent comorbidity (14.9%) followed by psychiatric illness with 12.7%. In both children and adults, the most represented type of TB was pulmonary TB (61.5% and 61.7%, respectively) followed by extrapulmonary TB with 23.1% in children and 22.2% in adults (Table 1).

**Table 1:** Descriptive demographic, epidemiological, and TB episode data

	Children		Adults		Total	
	*N* = 13	%	*N* = 81	%	*N* = 94	%
Sex						
Men	10	76.9	58	71.6	68	72.3
Women	3	23.1	23	28.4	26	27.6
Age, median (IQR)*	10 (7–12)		45 (34–57)		43 (27–55)	
< 18	13	100	-	–	13	13.8
18–40	–	–	32	39.5	32	47.8
41–60	–	–	34	40.7	34	35.1
> 60	–	–	16	19.7	16	17
Country of origin						
Spain	10	76.9	35	43.2	45	47.9
Out of Spain	3	23.1	46	56.8	49	52.1
Smoking habit						
Active smoker	–	–	32	40	32	34.4
Ex-smoker	–	–	11	13.7	11	11.8
Never smoked	13	100	37	46.2	50	53.7
Alcohol intake						
Daily or almost daily	–	–	15	19.7	15	16.8
Weekly	–	–	4	5.3	4	4.5
Monthly	–	–	5	6.6	5	5.6
Less than monthly	–	–	5	6.6	5	5.6
Never	13	100	47	61.8	60	67.4
Unknown	–	–	5	6	5	5.2
Comorbidities						
Diabetes	0	0	14	17.3	14	14.9
COPD	0	0	5	6.2	5	5.4
HIV	0	0	2	2.6	2	2.2
Renal disease	0	0	2	2.5	2	2.1
Cirrhosis	0	0	1	1.3	1	1.1
Psychiatric illness	1	7.7	11	13.6	12	12.7
Previous exposure to TB drugs						
No	13	100	73	91.2	86	92.5
Yes	0	0	7	8.7	7	7.5
Tuberculin history						
Negative	2	15.4	3	3.7	5	5.3
Positive	9	69.2	14	17.3	23	24.5
Never done	2	15.4	56	69.1	58	61.7
Unknown	0	0	8	9.9	8	8.5
Type of diagnosis						
Microbiologic	4	30.7	77	95	81	86.2
Clinic	9	69.2	4	4.9	13	13.8
Type of TB						
Pulmonary	8	61.5	50	61.7	58	61.7
Pulmonary + extrapulmonary	1	7.7	10	12.3	11	11.7
Extrapulmonary	3	23.1	18	22.2	21	22.3
Disseminated	1	7.7	3	3.6	4	4.3
Culture						
Negative	9	69.2	4	5.2	13	14.4
*M.tb* positive	4	30.7	70	90.9	74	82.2
Positive for other mycobacterium	0	0	1	1.3	1	1.1
Not done	0	0	2	2.6	2	2.2
AFB positive						
Pulmonary	1	100	28	77.7	29	78.3
Pulmonary + Extrapulmonary	0	0	3	8.3	3	8.1
Extrapulmonary	0	0	3	8.3	3	8.1
Disseminated	0	0	2	5.5	2	5.4
Drug resistance						
Sensible	2	16.7	61	81.3	63	72.4
MDR**	0	0.0	2	2.7	2	2.3
Monoresistance	2	16.7	6	8	8	9.2
Not done	8	66.7	6	8	14	16.1
Delay diagnostic (days)*** median (IQR)*	59 (16–79)		40 (21–81)		42 (19–81)	

*IQR* interquartile range, *MDR* multi-drug resistance

*IQR: Interquartile range; **MDR: Multi-drug resistance; ***Diagnostic delay: period of time between the onset of symptoms and diagnosis

We compared the results obtained with the last TB report issued by the Public Health Agency of Barcelona (2021) [19], confirming that our study population is representative of the TB patient population of the city of Barcelona.

The SARS-CoV-2 pandemic impacted recruitment and follow-up, with 84% of subjects being recruited before the pandemic (April 2018–March 2020). A total of nine individuals missed follow-up visits because of healthcare disruption.

### Clinical and microbiological data

Around 95.1% and 30.7% of adult and paediatric cases, respectively, were culture-positive, with 10.8% being monoresistant strains and 2.7% MDR-TB [determined by a susceptibility test (genotypic/phenotypic/both)] (Table 1).

**Fig. 1:**
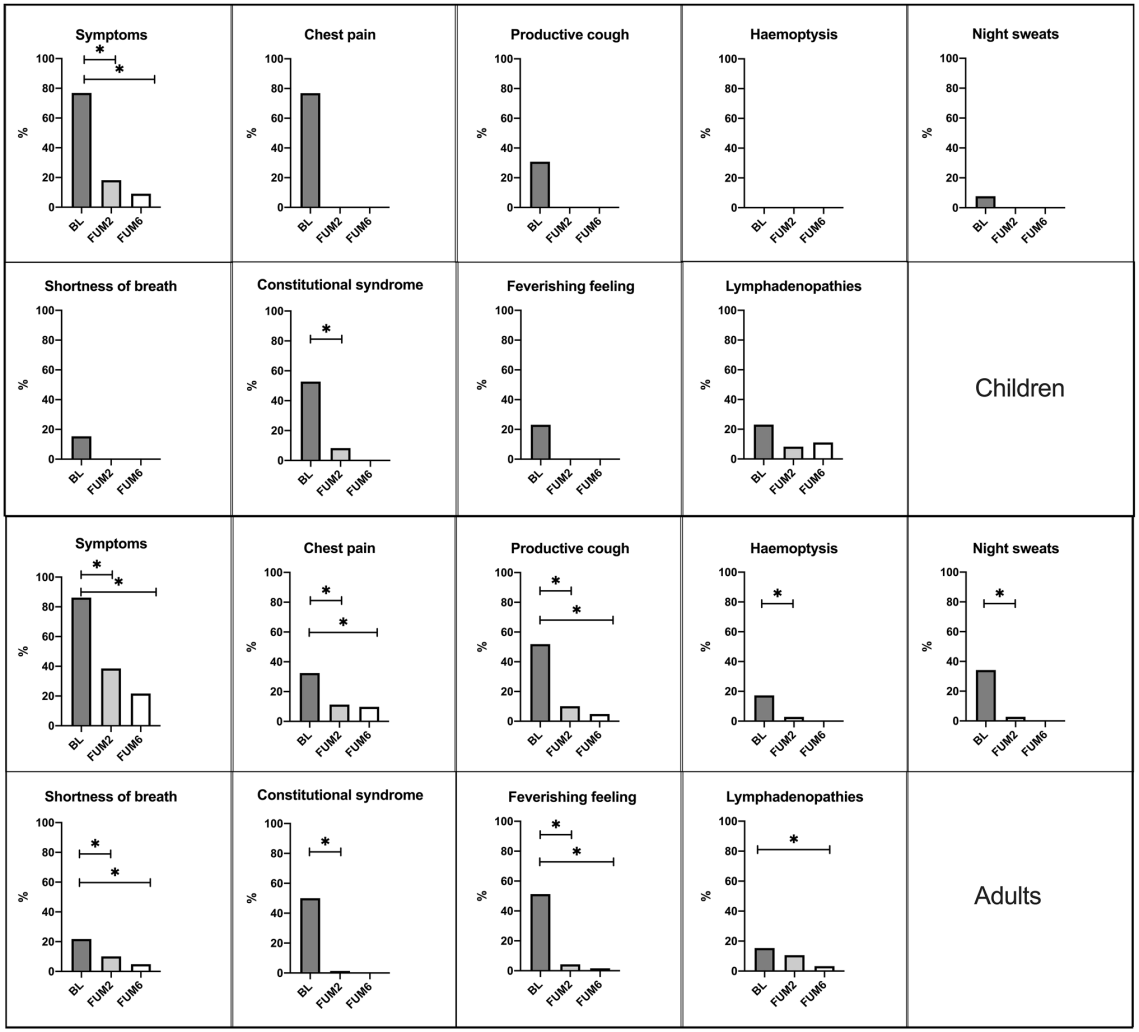
Evolution of symptoms in children and in adults. *p value < 0.05 (statistical test: Wilcoxon test for paired data)

A significant decrease in overall symptomatology was observed in individuals during treatment (*p* value < 0.05). In BL, the most prevalent symptoms were productive cough (51.9%), fever (51.3%) and constitutional syndrome (50%) in adults, and constitutional syndrome (53.8%), productive cough (30.8%) and fever and lymphadenopathy (23.1%) in children. In adults, a statistically significant decrease in all symptoms was observed by FUM2, even if a 21.7% still had symptoms by FUM6. These symptoms included chest pain, productive cough, dyspnoea, febrile sensation, and lymphadenopathy. Children’s symptoms tended to improve more rapidly, with only 1 child (9.1%) presenting symptoms by FUM6. At FUM6, all participants were considered cured. (Fig. 1).

Detailed data are appended in Supplementary Table S1.

A total of 58 (61.7%) participants had pulmonary TB, 29 (50%) had an acid-fast bacillus (AFB) in sputum smear and 48 (82.7%) had a positive sputum culture at BL. At FUM2, 15/21 had converted to negative AFB, and 18/25 had sputum culture conversion (SCC) to negative (fast converters).

### Inflammatory parameters

In adults, the CRP, ESR, NLR, and MLR levels were higher in those individuals with higher bacillary load (AFB, culture positivity) (*p* value < 0.05). Individuals presenting symptoms also had higher ESR. People with TB with lymphadenopathy had lower levels of CRP, NLR, and MLR than those without it. In addition, alcohol abusers had higher values of CRP, NLR, and MLR than the other people. Only the CRP level was affected by sex (*p*-value<0.05). The completed table with the IP values at BL is detailed in the Supplementary Table S2.

Overall, PI decreased significantly over time. In adults, MLR and CH50 levels did not decrease significantly until FUM6. For all other parameters (CRP, ESR, ESR, NLR, C3, and C4), these levels decreased significantly at FUM2 and FUM6. In children, a significant decrease in values was only observed for CRP, ESR, ESRV, NLR, and C4, and this did not occur for ESR and C4 until FUM6. CH50, C3, and C4 levels were observed to be within normal values in both adults and children at BL and during treatment. (Table 2).

**Table 2:** Evolution of the inflammatory parameters in children and in adults

	Children			Adults			Total		
	*n*	Median	IQR	*n*	Median	IQR	*n*	Median	IQR
C- reactive protein									
Baseline	11	4.3	0.9–66.4	71	7.41	1.32–16	82	6.4	1.32–16.4
FUM2	11	0.4*	0.2–9.8	54	1.67*	0.6–6.2	65	1.3*	0.45–6.4
FUM6	9	0.3*	0.2–2	51	0.42*$	0.19–2.4	60	0.4*$	0.2–2.2
ESR									
Basal	11	30	10–43	54	62.5	32–96	65	51	27–87
FUM2	10	9.5	5–20	51	42*	16–106	61	32*	10–79
FUM6	9	2*	2–7	47	25*$	12–57	56	20.5*$	8–52.4
Neutrophils/lymphocytes									
Basal	12	2.4	1.6–3.6	76	3.4	2.6–5	88	3.3	2.45–5
FUM2	11	1.5*	1.1–2.7	70	2.6*	1.8–3.4	81	2.5*	1.7–3.3
FUM6	9	0.9*	0.8–1.2	60	2.1*$	1.6–2.6	69	2*$	1.5–2.6
Monocytes/lymphocytes									
Basal	12	0.3	0.2–0.4	78	0.4	0.3–0.6	90	0.4	0.3–0.6
FUM2	12	0.2	0.2–0.2	70	0.4	0.3–0.5	82	0.3*	0.3–0.5
FUM6	9	0.2	0.2–0.2	60	0.3*$	0.2–0.4	69	0.3*$	0.2–0.4
CH50									
Basal	4	71	42–92.5	36	68.2	62.5–81.3	40	68.2	61.2–82
FUM2	9	56.1	52.8–73	32	70.6	58.9–87.1	41	69.9	57.8–79
FUM6	5	52	51–57	38	63.8*$	54.4–68.9	43	63.6*$	52–68.9
C3									
Basal	9	144	124.1–176	52	158	128–190.2	61	155.3	128–189
FUM2	11	123	108–147.1	47	147*	119–161	58	136.5*	119–157
FUM6	8	110.7	96.1–125	41	125*$	111–145	49	124*$	110–143.1
C4									
Basal	9	28.2	15.1–33.4	52	32.5	25.9–39.1	61	31.8	25–39
FUM2	11	24.6	18.5–35.2	47	28.6*	23.5–37	58	28*	23–35.2
FUM6	8	23.2*	15.9–26	41	25*$	21–31.4	49	24.1*$	20.3–31

*IQR* interquartile range

^*^p value < 0.05 when compared to the baseline visit (statistical test: Wilcoxon test for paired data)

^$^*p* value < 0,05 when compared to the 2-month follow-up visit (FUM2) (statistical test: Wilcoxon test for paired data)

*N* (*n*) shows the number of patients for which each variable was collected per each point

Different parameters influenced the evolution of the IP over time in adults and in children. The details are available in Supplementary Table S3.

### SGRQ results

A total of 85 completed SGRQs from 38 adults with pulmonary TB were analysed. At BL, the median SGRQ score was 28.1, progressing to 32.5 at FUM2 and 17.1 at FUM6, thus being above the normal range of 5–7 points (Table 3).

**Table 3:** Evolution of SGRQ total score and Kessler-10 scale

	Total score SGRQ							Kessler-10 scale						
	N1	Median BL (a)	Median FUM2 (b)	p value 1	N2	Median FUM6 (c)	*p* value 2	N1	Median BL (a)	Median FUM2 (b)	*p* value 1	N2	Median FUM6 (c)	*p* value 2
Total	22	28.1	32.5	0.8	19	17.1	0.004*	55	14	11	0.01*	49	10	0.001*
Sex														
Men	15	14.2	31.8	0.1	12	18.6	0.07	41	14	11	0.08	36	10	0.006*
Women	7	46.3	33.1	0.04*	7	12.9	0.03*	14	14	11	0.07	13	12	0.09
Age														
18–40	8	19.8	20.8	0.9	6	18.6	0.1	23	13	10	0.45	18	10	0.3
41–60	10	44.9	40.9	0.7	11	23	0.02*	21	20	12	0.009*	20	12,5	0.001*
> 60	4	5.3	7.1	0.7	2	4.1	0.6	11	10	10	0.7	11	0	0.2
Country of origin														
Spain	9	41	33.1	0.9	7	22.37	0.02*	21	20	12	0.03*	19	14	0.01*
Out of Spain	12	16.3	21.3	0.7	11	12.2	0.1	33	12	10	0.2	29	10	0.06
Alcohol habit														
Yes	8	23.6	32.3	0.2	6	18.7	0.05*	21	17	11	0.05*	18	11	0.05*
No	11	46.3	38.4	0.1	11	14	0.01	29	13	10	0.09	26	10	0.02*
Smoking habit														
Yes	11	44	44	0.2	9	20.3	0.04*	23	14	17	0.4	23	10	0.003*
No	10	9.64	9	0.1	10	12.55	0.04*	31	15	10	0.007*	25	10	0.09
Comorbidities														
Yes	4	5.3	14.5	0.7	3	20.3	0.3	13	10	14	0.3	13	10	0.6
No	18	35.8	35.75	0.7	16	15.55	0.01*	42	16,5	10	0.001*	36	10	0.0006*
Psychiatric disease														
Yes	2	60.7	55.4	0.2	2	46.39	0.7	6	28,5	16	0.52	5	0	0.06
No	20	23.55	32.1	0.9	17	14	0.003*	49	13	11	0.02*	44	10	0.006*
CRP														
< 5 mg/L	10	17.95	35.1	0.9	8	23.4	0.3	20	17,5	11	0.05*	18	10	0.06
> 5 mg/L	8	43.5	44.6	0.7	9	14	0.01*	27	13	11	0.2	24	10	0.05*
ESR														
< 20 mm/h	3	7	7.2	1	1	20.1	0.3	3	13	13	0.8	2	8,5	0.6
> 20 mm/h	14	38.5	41.6	0.6	14	19.7	0.01*	31	17	12	0.04*	30	10	0.0007*
AFB														
Positive	13	41	38.4	0.8	13	14	0.005*	29	17	18	0.3	26	10	0.002*
Negative	9	10.8	8.48	0.4	6	23.4	0.6	21	14	10	0.009*	19	10	0.08*
Culture														
Positive	21	25.4	32.4	0.9	18	15.55	0.007*	52	14	10.5	0.02*	48	10	0.002*
Negative	0	–	–	–	–	–	–	0	–	–	–	–	–	–
Symptoms														
No	3	6.2	8.48	0.6	2	26.39	0.2	7	12	0	0.1	6	15	0.7
Yes	19	40.9	33.1	0.6	17	14	0.0007*	48	14,5	11.5	0.03*	43	10	0.0007*
Chest pain														
No	18	26.2	32.1	0.8	13	14	0.05*	37	14	11	0.06	34	10	0.01*
Yes	4	33.2	38.1	0.9	6	18.7	0.03*	16	16	12	0.3	14	10	0.05*
Productive cough														
No	9	14.2	19	0.8	7	20.3	0.6	21	12	8	0.04*	20	12	0.15
Yes	13	46.3	33.1	0.6	12	12.5	0.003*	33	15	11	0.16	28	10	0.004*
Haemoptysis														
No	16	26.2	32.6	0.6	16	20.2	0.01*	39	13	11	0.01*	35	12	0.01*
Yes	5	46.6	39.8	0.8	2	9.2	0.2	12	18,5	11.5	0.7	10	10	0.2
Night sweets														
No	12	23.6	35.1	0.3	10	18.6	0.2	33	13	10	0.1	30	10	0.1
Yes	10	41	32.6	0.08	9	12.9	0.008*	19	17	12	0.06*	16	11,5	0.008*
Shortness breath														
No	17	21.7	31.8	0.7	15	14	0.03*	41	13	10	0.05*	37	10	0.02*
Yes	5	48.7	43.4	0.3	4	23.5	0.07	12	20	15.5	0.3	11	10	0.02*
Constitutional syndrome														
No	12	14.6	9.9	0.5	8	18.6	0.5	24	14	10.5	0.2	22	10	0.07
Yes	10	43.7	35.8	0.2	10	17.1	0.007*	29	15	12	0.05*	26	10	0.009*
Feverish feeling														
No	12	13.7	14.5	0.8	8	21.5	0.4	24	16	11.5	0.02*	21	13	0.08
Yes	10	43.7	38.6	0.8	11	14	0.003*	30	14	10.5	0.2	27	10	0.005*

*BL* baseline visit, *FUM2* follow-up month 2 visit, *FUM6* follow-up month 6 visit, *p value 1* *p* value between BL and FUM2, *p value 2* *p* value between BL and FUM6, *N1* Number of participants with SGRQ/Kessler-10 completed in BL and FUM2, *N2* number of participants with SGRQ/Kessler-10 completed in BL and in FUM6

(a): median of the BL SGRQ/Kessler-10 in participants with SGRQ/Kessler-10 completed in BL and in FUM2

(b): median of the FUM2 SGRQ/Kessler-10 in participants with SGRQ/Kessler-10 completed in BL and in FUM2

(c): median of the FUM6 SGRQ/Kessler-10 in participants with SGRQ/Kessler-10 completed in BL and in FUM6

^*^p value < 0.05 when compared to the baseline visit (statistical test: Wilcoxon test for paired data)

Those participants older than 60 years scored within the normal range during treatment, and a statistically significant decrease was only observed for females. When comparing SGRQ values at BL to those at FUM6, SGRQ values decreased significantly in middle-aged participants, participants with no comorbidities, participants from Spain, those with CRP ≥ 5 mg/L or ESR ≥ 20 mm/h, participants with a positive culture, and those presenting productive cough or systemic symptoms (Table 3).

### Kessler-10

We collected 164 Kessler-10 questionnaires from 60 individuals. From these 60 participants, BL and FUM2 paired data were available for 55 individuals. The median score for our cohort was 14 at BL, 11 at FUM2, and 10 at FUM6. People aged 41–60 years, born in Spain, and those with a psychiatric disease history scored at BL higher than the cut-off provided by the developers of the questionnaire (20 points) at BL; additional detail is provided in Supplementary Table S4. A significant improvement was observed at both FUM2 and FUM6 (Table 3).

### Indicators to predict TB improvement

We first assessed whether there were any differences in terms of IPs, bacillary load, clinical severity, SGRQ total score, and Kessler-10 score levels at BL when comparing the slow and the fast sputum culture converters, considering only the data from pulmonary, microbiologically confirmed TB cases. Clinical severity (≥ 4 symptoms) predicted the slower SCC (OR: 8.1, *p* = 0.004; Table 4a). With regard to predicting the SCC at FUM2 using ROC curves, better results were achieved with CRP [AUC = 0.716, cut-off ≥ 0.82 mg/L, sensitivity (*S*) = 85%, specificity (*E*) = 22.2%]. The rest of results are detailed in the Supplementary Table S5a and the ROC curves representation are included in the Supplementary material 6a.

**Table 4:** Analysis of the culture conversion and presence of symptoms at FUM2

(a) Analysis of predictive factors for the culture conversion at month 2 (only cases culture + at BL)							
	N/Total	%	N/CC FUM2^a^	%	OR	IC	*p* value^c^
CRP Cat (> 5 mg/L)	17/29	58.60	11/20	55.00	0.62	0.12–3.26	0.6
ESR cat (> 20 mm/h)	25/27	93.00	17/18	94.40	1.98	0.11–36.59	0.6
NLR_cat ^d^	19/31	61.30	13/22	59.10	0.78	0.15–4.10	0.8
MLR_cat ^d^	20/31	64.50	14/22	63.60	0.86	0.16–4.52	0.8
C3_cat^d^	14/26	53.80	11/20	55.00	1.24	0.19–7.97	0.8
C4_cat^d^	15/26	57.70	11/20	55.00	0.61	0.09–4.13	0.6
CH50_cat^d^	14/23	60.90	11/17	64.70	1.74	0.25–12.24	0.6
AFB	8/31	25.80	5/22	22.70	0.57	0.11–3.20	0.5
*Clinical severity^e^	13/25	52.00	11/16	68.75	**8.1**	**1.13–56.47**	**0.04**
Total score SGRQ_cat^f^	17/20	85.00	10/13	76.90	–	–	0.3
Kessler-10_cat^g^	8/20	32.00	6/18	33.30	0.97	0.13–7.38	0.9

(b) Analysis of predictive factors for the presence of symptoms at month 2 (only cases with symptoms at BL)							
	N/Total	%	N/PSFUM2^b^	%	OR	IC	*p* value^c^
CRP Cat (> 5 mg/L; < 5 mg/L)	35/60	58.30	14/22	63.60	1.48	0.50–4.43	0.5
ESR cat (> 20 mm/H; < 20 mm/h)	41/47	87.20	17/17	100.00	–	–	**0.05**
NLR_cat^d^	31/65	47.70	10/24	41.70	0.67	0.24–1.87	0.4
MLR_cat^d^	32/65	49.20	11/23	47.80	0.88	0.31–2.47	0.8
C3_cat^d^	23/45	51.10	10/16	62.50	1.93	0.54–6.85	0.3
C4_cat^d^	23/45	51.10	12/16	75.00	**4.88**	**1.23–19.30**	**0.024**
CH50_cat^d^	16/32	50.00	7/12	58.30	1.72	0.40–7.37	0.5
AFB	34/64	53.10	7/23	30.40	**0.23**	**0.07–0.68**	**0.009**
Culture	54/64	84.40	21/24	87.50	1.45	0.29–7.18	0.6
*Clinical severity^e^	23/45	51.11	11/19	57.89	1.05	0.71–1.55	0.8
Total score SGRQ^f^	28/34	82.30	13/14	92.80	4.98	0.45–54.66	0.2
Kessler-10_catg	17/42	40.50	7/16	43.70	0.97	0.13–7.38	0.9

Bold depicts statistically significant differences

^a^CCFUM2: culture conversion at FUM2

^b^PSFUM2: presence of symptoms at FUM2

^c^adjusted by age

^d^categories: ≥ median; < median

^e^categories: ≥ 4 symptoms; < 3 symptoms

^f^categories: ≥ 7; < 7

^g^categories: ≥ 20; < 20

We found significantly differences according to ESR levels higher than 20 mm/h, C4 values above the BL median (32.55 mg/dl), and AFB positivity at BL (Table 4b). With regard to predicting the presence of symptoms at FUM2, better AUC results were achieved for ESR (AUC = 0.6154, cut-off ≥ 47 mm/h, *S* = 88.24%, *E* = 40%); C4 (AUC = 0.6437, cut-off ≥ 32.7 mg/dl, *S* = 75%, *E* = 62.07%); and SGRQ total score (AUC = 0.6395, cut-off ≥ 25.4; *S* = 71.43, *E* = 60). The rest of results are detailed in the Supplementary Table S5b and the ROC curves’ representation is included in the Supplementary material 6b).

## Discussion

In this study, we followed up a longitudinal prospective cohort of 94 TB people from five hospitals in Barcelona between 2018 and 2021 and analysed the ability of several variables to predict TB improvement. The demographic characteristics of our cohort align with findings from other retrospective cohorts in our region [20].

Our results showed that most IPs tend to decrease during treatment. Despite this, clinical severity, but not IPs, was associated with SCC at 2 months. The best predictor was CRP although with very low specificity. We analysed the IPs up to FUM6, to determine their usefulness in monitoring TB evolution and response to treatment, as we could only find one European cohort extending up to the end of treatment [12], and that study only assessed CRP and ESR. The CRP results up to the end of intensive treatment are in line with those in the literature [21, 22], and showed a decrease up to FUM6, thus suggesting that they could be useful for monitoring disease evolution. A decrease in IPs levels has been described in those individuals who achieved culture conversion [23]. Additionally, our results, in line with others [24], showed that individuals with microbiologically confirmed TB and those with higher bacillary load had higher IPs levels at baseline, thus suggesting a link between IPs levels and disease severity. As far as we are aware there are no other TB cohorts assessing the relationship between IPs and symptoms. In our cohort, the decrease in symptomatology and IPs values over time indicates that these parameters may be related to disease severity, and that sustained high levels may be associated with lower treatment efficacy. Based on our results, clinicians could use ESR values to warn individuals with higher levels about the persistence of potential symptoms at FUM2, thus helping them better cope with the expected outcomes. In terms of treatment response, clinical severity, measured as presence of ≥ 4 symptoms, better predicted slower culture conversion.

In our study, we evaluated the evolution of symptoms and IP in 13 children. In paediatric TB, the age group under 5 years is the one that differs most from adults as, in our setting, TB diagnosis is usually achieved early and in the context of contact tracing, and younger children tend to develop disseminated TB rather than pulmonary forms [25]. Our study included children (median age: 10 years) who exhibited similarities to adults but demonstrated distinct differences in symptom frequency. As for adults, CRP, ESR, and neutrophil count decreased during follow-up. However, limited sample sizes in paediatric studies highlight the need for further research in this population.

People with TB in our cohort did not fully recover lung functionality, especially those presenting symptoms or psychiatric comorbidities. Despite microbiological cure, TB can impact people’s lives beyond the end of treatment as it can leave permanent tissue damage [26]. The lack of sufficient data on Post-TB Lung Disease (PTLD) is a challenge in TB management, as it directly impacts people’s QoL [27]. Assessing the impact of TB on lung function is not commonly performed as routine practice [28]. We used the SGRQ as a proxy of self-perceived functional impairment, as it was designed to measure the impaired health and perceived HRQoL in airways disease. Even if not specific for TB, this questionnaire has been widely used in TB clinical studies and trials but not routinely used in clinical settings [10]. Unlike certain studies conducted outside of Europe [17, 25] which reported an improvement in the total SGRQ score during the intensive treatment phase, we did not observe the same outcome in our cohort. Our findings, similar to others [29–31], revealed significant improvement after 6 months of treatment, but residual functional impairment was still reported. These findings collectively suggest that the pulmonary impact of TB may be underappreciated in patient management and its screening should be encouraged in order to implement measures when needed.

Finally, there is continuously increasing evidence that links mental health impairment and TB, and that integrated mental health care is needed in TB population [32, 33]. We observed an overall and early improvement in the total measured score throughout the treatment period for the entire cohort. The Kessler-10 or other, more specific scales could be used as an initial screening for all individuals and, if scoring abnormally and considered appropriate, they could then be referred for further psychological and/or psychiatric assessment, follow-up, and specific interventions.

Our study has some limitations. First, the biomarkers analysed lack specificity to TB, and the results may have been influenced by other variables, such as comorbidities or not conducting sample analysis in a single central laboratory. However, these facts may make our results more representative of a real-life scenario. Additionally, the sample size, particularly for paediatric cases, limits the statistical power of the analysis. Finally, we obtained more participants with Kessler-10 than SGRQ paired data, what we do consider is related to the length of the questionnaires. While the Kessler-10 is only 10-questions long, the SGRQ includes 76 questions, requiring more time to be completed and inducing more fatigue. This must be considered when selecting the appropriate tool to measure impact on HRQoL.

## Conclusion

In conclusion, this study analyses the evolution of IPs, symptoms and HRQoL up to month 6, and the relationship between them, in a prospective cohort in the WHO European region. We have seen that IP levels at BL are related to the bacillary load and TB severity and can help us to predict the permanence of symptoms later on. Large-scale studies are required to determine the applicability of these findings, but our recommendation would be to include CRP and ESR assessment for improved clinical follow-up of people with TB.

Additionally, there are currently no programmes aimed at assessing and monitoring the HRQoL of TB people. We have confirmed that there is PTLD at the end of treatment and groups of TB people with above-normal levels of psychological distress. Our research underscores the significance of implementing health strategies for assessing both lung function and mental health across the entire disease progression. This proactive approach allows us to identify any potential risks early on. Subsequently, we can tailor interventions—ranging from pharmacological to non-pharmacological methods such as pulmonary rehabilitation or referrals for further psychiatric assessment. These interventions aim to significantly enhance people's quality of life and play a pivotal role in facilitating their complete recovery.

### Supplementary Information

Below is the link to the electronic supplementary material.

Supplementary file1 (DOCX 358 KB)

## Data Availability

Data supporting the findings of this study are accessible within the paper and its Supplementary Information. However, individual patient data is currently unavailable to third parties. This is due to the fact that providing access would require a distinct data processing procedure from that outlined in the informed consent. The data are securely stored in controlled access storage at the IGTP.
