# Supplementary material for: A longitudinal prospective study of active tuberculosis in a Western Europe setting: insights and findings
Source: Infection. 2024 Feb 13;52(2):611–23. doi: 10.1007/s15010-024-02184-2 (PMC10954962; doi:10.1007/s15010-024-02184-2)
Supplement: Supplementary file 1 — Supplementary file1 (DOCX 358 KB) [file 15010_2024_2184_MOESM1_ESM.docx]

Supplemmentary material

Table S1: Evolution of symptoms in children and in adults

|  |  | | **Children** | | | | | | **Adults** | | | | | | **Total** | | | | | |
| --- | --- | --- | --- | --- | --- | --- | --- | --- | --- | --- | --- | --- | --- | --- | --- | --- | --- | --- | --- | --- |
|  |  | |  | | **N** | | **n** | | **%** | | **N** | | **n** | | **%** | | **N** | | **n %** | |
| **Symptoms** | | **Basal** | | 13 | | 10 | | 76,9 | | 80 | | 69 | | 86,2 | | 93 | | 79 | | 84,9 |
|  |  | **FUM2** | | 11 | | 2 | | 18,2* | | 65 | | 25 | | 38,5* | | 76 | | 27 | | 35,5* |
|  |  | **FUM6** | | 11 | | 1 | | 9,1* | | 60 | | 13 | | 21,7* | | 71 | | 14 | | 19,7* |
| **Chest pain** | | **Basal** | | 13 | | 2 | | 15,4 | | 77 | | 25 | | 32,47 | | 90 | | 27 | | 30 |
|  |  | **FUM2** | | 12 | | 0 | | 0 | | 71 | | 8 | | 11,3* | | 83 | | 8 | | 9,64* |
|  |  | **FUM6** | | 11 | | 0 | | 0 | | 61 | | 6 | | 9,8* | | 72 | | 6 | | 8,3* |
| **Productive cough** | | **Basal** | | 13 | | 4 | | 30,8 | | 79 | | 41 | | 51,9 | | 92 | | 45 | | 48,9 |
|  |  | **FUM2** | | 12 | | 0 | | 0 | | 70 | | 7 | | 10* | | 82 | | 7 | | 8,5* |
|  |  | **FUM6** | | 11 | | 0 | | 0 | | 61 | | 3 | | 4,9* | | 72 | | 3 | | 4,2* |
| **Haemoptysis** | | **Basal** | | 13 | | 0 | | 0 | | 75 | | 13 | | 17,3 | | 88 | | 13 | | 14,8 |
|  |  | **FUM2** | | 12 | | 0 | | 0 | | 59 | | 2 | | 2,9* | | 83 | | 2 | | 2,4* |
|  |  | **FUM6** | | 11 | | 0 | | 0 | | 61 | | 0 | | 0 | | 72 | | 0 | | 0 |
| **Night sweats** | | **Basal** | | 13 | | 1 | | 7,7 | | 76 | | 26 | | 34,2 | | 89 | | 27 | | 30,3 |
|  |  | **FUM2** | | 12 | | 0 | | 0 | | 71 | | 2 | | 2,8* | | 83 | | 2 | | 2,4* |
|  |  | **FUM6** | | 11 | | 0 | | 0 | | 61 | | 0 | | 0 | | 72 | | 0 | | 0 |
| **Shortness of breath** | | **Basal** | | 13 | | 2 | | 15,4 | | 78 | | 18 | | 21,8 | | 91 | | 19 | | 20,9 |
|  |  | **FUM2** | | 12 | | 0 | | 0 | | 70 | | 7 | | 10* | | 82 | | 7 | | 8,5* |
|  |  | **FUM6** | | 11 | | 0 | | 0 | | 61 | | 3 | | 4,9* | | 72 | | 3 | | 4,2* |
| **Constitutional syndrome** | | **Basal** | | 13 | | 7 | | 53,8 | | 76 | | 38 | | 50 | | 89 | | 45 | | 50,6 |
|  |  | **FUM2** | | 12 | | 1 | | 8,3* | | 70 | | 1 | | 1,4* | | 82 | | 2 | | 2,4* |
|  |  | **FUM6** | | 11 | | 0 | | 0 | | 61 | | 0 | | 0 | | 72 | | 0 | | 0* |
| **Feverish feeling** | | **Basal** | | 13 | | 3 | | 23,1 | | 78 | | 40 | | 51,3 | | 91 | | 43 | | 47,2 |
|  |  | **FUM2** | | 11 | | 0 | | 0 | | 69 | | 3 | | 4,3* | | 80 | | 3 | | 3,7* |
|  |  | **FUM6** | | 11 | | 0 | | 0 | | 61 | | 1 | | 1,6* | | 72 | | 1 | | 1,4* |
| **Lymphadenopathies** | | **Basal** | | 13 | | 3 | | 23,1 | | 78 | | 12 | | 15,4 | | 90 | | 14 | | 15,6 |
|  |  | **FUM2** | | 12 | | 1 | | 8,3 | | 66 | | 7 | | 10,6 | | 78 | | 8 | | 10,3 |
|  |  | **FUM6** | | 11 | | 1 | | 11,1 | | 60 | | 2 | | 3,3* | | 68 | | 3 | | 4,2* |
| N: total number of patients with complete information on this symptom at the visit | | | | | | | | | | | | | | | | | | |  | |
| n: number of patients with symptom | | | | | | |  | |  | |  | |  | |  | |  | |  | |
| * p-value<0,05 when compared to the baseline visit (statistical test: Wilcoxon-test for paired data) $ p-value<0,05 when compared to the two-month follow-up visit (FUM2) (statistical test: Wilcoxon-test for paired data) | | | | | | | | | | | | | | | | | | | | |

Table S2: Differences in the inflammatory parameter’s values at baseline in children and adults.

|  | **Baseline** | | | | | | | | |  |  |  |
| --- | --- | --- | --- | --- | --- | --- | --- | --- | --- | --- | --- | --- |
|  | **CRP** | | | **ESR** | | | **NLR** | | | **MLR** | | |
|  | **n** | **median** | **IQR** | **n** | **median** | **IQR** | **n** | **median** | **IQR** | **n** | **median** | **IQR** |
| **Sex** |  |  |  |  |  |  |  |  |  |  |  |  |
| Men | 51 | 9,8 | (1,9-24,5) | 38 | 51,5 | (20-98) | 55 | 3,5 | (2,6-5,1) | 55 | 0,5 | (0,3-0,8) |
| Women | 20 | **1,4*** | (0,8-6,2) | 16 | 70,5 | (49,7-98) | 21 | 3,2 | (2,5-3,8) | 23 | 0,3 | 0,3-0,6) |
| **Age** |  |  |  |  |  |  |  |  |  |  |  |  |
| 18 – 40 | 28 | 7,1 | (1,5-16,2) | 19 | 47 | (20-95) | 29 | 3,1 | (2,0-4) | 30 | 0,4 | (0,3-0,6) |
| 41 – 60 | 32 | 5,7 | (1,1-13,1) | 27 | 71 | (48-120) | 32 | **3,9*** | (2,7-5,8) | 33 | 0,5 | (0,3-0,8) |
| > 60 | 11 | 16 | (1,2-30,6) | 8 | 42,5 | (25,5-80,75) | 15 | 3,2 | (2,5-4,2) | 15 | 0,4 | (0,2-0,7) |
| **Country of origin** |  |  |  |  |  |  |  |  |  |  |  |  |
| Spain | 30 | 8,8 | (1-29,9) | 26 | 68,5 | (29-96,7) | 42 | 3,2 | (2,1-4,9) | 34 | 0,5 | (0,4-0,8) |
| Out of Spain | 40 | 5,7 | (1,6-15,9) | 27 | 56 | (32-104) | 33 | 3,6 | (2,7-5,5) | 44 | 0,35 | (0,3-0,6) |
| **Tobacco** |  |  |  |  |  |  |  |  |  |  |  |  |
| Never smoke | 32 | 2,1 | (0,8-14,5) | 23 | 55 | (11-72) | 33 | 2,4 | (1,5-4) | 35 | 0,4 | (0,3-0,6) |
| Smoke or exsmoker | 39 | 8,93 | (1,93-24,5) | 31 | 72 | (42-108) | 42 | 3,5 | (2,7-5,1) | 42 | 0,5 | (0,3-0,8) |
| **Alcohol** |  |  |  |  |  |  |  |  |  |  |  |  |
| Never | 42 | 3,6 | (1,1-3,6) | 33 | 70 | (32-97) | 44 | 3,3 | (2,5-5) | 45 | 0,4 | (0,3-0,6) |
| Some times | 26 | **14,6*** | (1,9-32,2) | 19 | 68 | (42-104) | 27 | **4,0*** | (2,9-6,9) | 28 | **0,5*** | (0,3-1) |
| **Comorbidities prior to TB** |  |  |  |  |  |  |  |  |  |  |  |  |
| No | 35 | 5,4 | (0,8-10,7) | 27 | 72 | (36-120) | 36 | 3,6 | (2,8-5,1) | 37 | 0,5 | (0,3-0,7) |
| Yes | 24 | 7,6 | (1,2-23,1) | 19 | 68 | (42-96) | 28 | 3,1 | (2,5-4,9) | 29 | 0,4 | (0,2-0,6) |
| **Previous exposure to TB drugs** |  |  |  |  |  |  |  |  |  |  |  |  |
| No | 65 | 7,4 | (1,3-16,2) | 50 | 68,5 | (32-100,2) | 68 | 3,6 | (2,7-5,1) | 70 | 0,5 | (0,3-0,7) |
| Yes | 5 | 2 | (0,6-25,3) | 4 | 26 | (5,5-72,7) | 7 | **1,9*** | (1,5-2,5) | 7 | **0,3*** | 0,2-0,4) |
| **Diagnosis** |  |  |  |  |  |  |  |  |  |  |  |  |
| Microbiologic | 60 | 8,6 | (1,5-18,3) | 45 | 56 | (29,5-91) | 65 | 3,5 | (2,5-5) | 66 | 0,5 | (0,3-0,6) |
| Clinic | 11 | 1,5 | (0,4-15,3) | 9 | 78 | (51,5-120) | 11 | 3,1 | (2,6-6,5) | 12 | 0,3 | (0,3-0,8) |
| **Type of tuberculosi** |  |  |  |  |  |  |  |  |  |  |  |  |
| Pulmonar | 43 | 6 | (1,3-15,8) | 34 | 68 | (42-120) | 48 | 3,5 | (2,8-5,3) | 47 | 0,5 | (0,3-0,8) |
| Pulmonar + Extrapulmonar | 9 | **16,4*** | (12,2-29,7) | 3 | 72 | (71-108) | 10 | 3,8 | (2,-5,5) | 10 | 0,5 | (0,4-0,8) |
| Extrapulmonar | 18 | 1,3 | (0,4-8,9) | 15 | 47 | (20-71) | 17 | 2,6 | (2,1-3,1) | 18 | 0,3 | (0,3-0,4) |
| Diseminated | 3 | 10,9 | (10,5-128,8) | 3 | 32 | (14-85) | 3 | 4,1 | (3,2-5,0) | 3 | 0,5 | (0,5-0,6) |
| **Culture** |  |  |  |  |  |  |  |  |  |  |  |  |
| Negative | 4 | 0,8 | (0,2-4,3) | 4 | 63 | (40,7-76,2) | 4 | 2 | (1,3-5,5) | 4 | 0,3 | 0,2-0,7) |
| Positive | 62 | **7,6*** | (1,4-16,1) | 47 | 69 | (27-104) | 67 | 3,5 | (2,6-5,1) | 70 | 0,4 | (0,3-0,7) |
| **Diagnosis delay** |  |  |  |  |  |  |  |  |  |  |  |  |
| < 42 days (6 weeks) | 25 | 3 | (1,5-20,2) | 20 | 70 | (29-98,2) | 27 | 2,9 | (2,4-5,0) | 27 | 0,5 | (0,3-0,5) |
| ≤ 42 days (6 weeks) | 23 | 5,4 | (0,9-9,4) | 20 | 75 | (44,7-117) | 23 | 3,7 | (3,1-6,5) | 26 | 0,4 | (0,3-0,7) |
| **AFB** |  |  |  |  |  |  |  |  |  |  |  |  |
| Negative | 29 | 1,4 | (0,5-15,9) | 24 | 47,5 | (17-71) | 35 | 2,7 | (2-4,1) | 35 | 0,4 | (0,2-0,6) |
| Positive | 34 | **8,3*** | (1,9-14,3) | 27 | **85*** | (50-120) | 33 | **3,7*** | (3,2-5,7) | 35 | **0,5*** | (0,3-0,7) |
| **BMI** |  |  |  |  |  |  |  |  |  |  |  |  |
| Low weight | 10 | 6 | (1,8-93,2) | 6 | 114 | (96-120) | 9 | 5,1 | (3,7-11,8) | 9 | 0,6 | (0,4-1,3) |
| Normal weight | 38 | 6 | (1,4-14,3) | 28 | **70*** | (34-97) | 35 | **3,4*** | (2,6-4,9) | 35 | 0,4 | (0,3-0,6) |
| Over weight | 18 | 3,4 | (0,2-13,6) | 13 | **55*** | (36-78) | 19 | **2,8*** | (1,8-4,0) | 19 | 0,4 | (0,2-0,6) |
| **Symptoms** |  |  |  |  |  |  |  |  |  |  |  |  |
| No | 10 | 6,2 | (0,5-54,5) | 9 | 32 | (9,5-45) | 10 | 4 | (2,6-4,4) | 11 | 0,4 | (0,2-0,6) |
| Yes | 61 | 7,4 | (1,4-15,9) | 45 | **71*** | (42-106) | 66 | 3,4 | (2,6-5,1) | 67 | 0,4 | (0,3-0,7) |
| **Chest pain** |  |  |  |  |  |  |  |  |  |  |  |  |
| No | 48 | 5 | (1,1-14,5) | 36 | 54 | (28,2-102) | 49 | 3,7 | (2,3-5,1) | 52 | 0,4 | (0,3-0,6) |
| Yes | 22 | 10,2 | (1,2-18) | 17 | 71 | (39,5-93) | 24 | 3 | (2,6-4,7) | 23 | 0,5 | (0,3-0,8) |
| **Productive cough** |  |  |  |  |  |  |  |  |  |  |  |  |
| No | 34 | 3,5 | (0,5-17,7) | 25 | 36 | (12,5-69) | 36 | 3,2 | (2,1-4,1) | 38 | 0,4 | (0,2-0,6) |
| Yes | 36 | 7,6 | (1,8-15,8) | 28 | **75*** | (50,7-116) | 39 | **3,5*** | (2,8-5,9) | 39 | **0,5*** | (0,3-0,8) |
| **Hemoptysis** |  |  |  |  |  |  |  |  |  |  |  |  |
| No | 56 | 6,8 | (1,7-18,2) | 44 | 56,5 | (32-95,7) | 59 | 3,5 | (2,6-4,9) | 61 | 0,4 | (04,-0,6) |
| Yes | 10 | 2,2 | (1,9-9,7) | 6 | 47,5 | (12,5-120) | 12 | 3,1 | (1,9-5,8) | 12 | 0,3 | (0,2-0,8) |
| **Night sweets** |  |  |  |  |  |  |  |  |  |  |  |  |
| No | 45 | 4,3 | (0,8-16) | 32 | 45 | (17-85) | 48 | 3,2 | (2,4-4,8) | 50 | 0,4 | (0,3-0,6) |
| Yes | 22 | 7,6 | (1,7-12,7) | 19 | **72*** | (53-99) | 24 | 3,5 | (2,6-6,2) | 24 | **0,5*** | (0,3-0,8) |
| **Shortness of breath** |  |  |  |  |  |  |  |  |  |  |  |  |
| No | 54 | 6,8 | (1,4-15,4) | 40 | 68,5 | (28,2-102) | 58 | 3,5 | (2,4-5,1) | 60 | 0,4 | (0,3-0,6) |
| Yes | 15 | 3 | (1,2-35,7) | 13 | 50 | (37-89) | 16 | 3,4 | (2,6-5,6) | 16 | 0,5 | (0,3-0,8) |
| **Constitutional syndrome** |  |  |  |  |  |  |  |  |  |  |  |  |
| No | 33 | 1,9 | (0,6-10) | 28 | 42 | (14,5-71,5) | 36 | 2,9 | (2,1-4,1) | 38 | 0,3 | (0,2-0,5) |
| Yes | 34 | **10,8*** | (3,9-18) | 25 | **79*** | (54-120) | 36 | **3,9*** | (2,8-5,5) | 36 | **0,5*** | (0,4-0,8) |
| **Fever feeling** |  |  |  |  |  |  |  |  |  |  |  |  |
| No | 34 | 2,5 | (0,6-16,1) | 26 | 39 | (15,5-68,5) | 36 | 3,2 | (2,2-4,2) | 38 | 0,4 | (0,2-0,6) |
| Yes | 35 | 9,4 | (1,8-24,5) | 27 | **79*** | (56-120) | 38 | 3,5 | (2,8-5,2) | 38 | 0,5 | (0,3-0,7) |
| **Lymphadenopathy** |  |  |  |  |  |  |  |  |  |  |  |  |
| No | 59 | 7,8 | (1,5-22,9) | 45 | 71 | (42-101,5) | 64 | 3,6 | (2,7-5,1) | 65 | 0,5 | (0,3-0,5) |
| Yes | 11 | **1,4*** | (0,2-12,2) | 8 | 29,5 | (13,2-66,2) | 11 | **2,1*** | (1,3-3,1) | 12 | **0,2*** | (0,2-0,4) |
| * p-value<0,05 when compared between groups (statistical test: Mann-Whitney test) | | | | | | | |  |  |  |  |  |

Table S3: Analysis of the influence of demographic, microbiological and clinical variables in the evolution of the IP over time in adults and in children.

RCP

|  |  |  | **CHILDREN** | | | **ADULTS** | | |
| --- | --- | --- | --- | --- | --- | --- | --- | --- |
|  |  |  | **Baseline** | **FUM2** | **FUM6** | **Baseline** | **FUM2** | **FUM6** |
| **Sex** | Men | **n** | 9 | 10 | 8 | 53 | 40 | 35 |
|  |  | **Median (IQR)** | 4,3 (65,5) | **0,33* (21,11)** | **1,3$ (2,93)** | 9,39 (17,05) | **3,79* (6,93)** | **1,05*$ (3,27)** |
|  | Women | **n** | 3 | 2 | 2 | 20 | 16 | 16 |
|  |  | **Median (IQR)** | 1,7 (28,16) | 3,9 (7,55) | 0,17 (0,07) | 1,38 (5,34) | **0,81* (1,33)** | **0,35*$ (0,5)** |
| **Age** | <18 | **n** | 12 | 12 | 10 | 0 | 0 | 0 |
|  |  | **Median (IQR)** | 3,3 (46,78) | **0,33* (15,35)** | **0,45* (2,76)** | - (-) | - (-) | - (-) |
|  | 18 - 40 | **n** | 0 | 0 | 0 | 29 | 24 | 19 |
|  |  | **Median (IQR)** | - (-) | - (-) | - (-) | 8,4 (10,3) | **2,80* (5,81)** | **0,56 *$ (1,39)** |
|  | 41    – 60 | **n** | 0 | 0 | 0 | 33 | 25 | 25 |
|  |  | **Median (IQR)** | - (-) | - (-) | - (-) | 5,37 (9,54) | **1,95* (3,69)** | **0,42*$ (0,85)** |
|  | > 60 | **n** | 0 | 0 | 0 | 11 | 7 | 7 |
|  |  | **Median (IQR)** | - (-) | - (-) | - (-) | 16 (29,44) | 2,05 (73,17) | 8,6 (16,63) |
| **Smoking habit** | Never smoked | **n** | 0 | 0 | 0 | 45 | 37 | 32 |
|  |  | **Median (IQR)** | - (-) | - (-) | - (-) | 2,3 (11,3) | **1,03* (6,1)** | **3,38*$ (1,39)** |
|  | Ex-smoker | **n** | 0 | 0 | 0 | 10 | 8 | 8 |
|  |  | **Median (IQR)** | - (-) | - (-) | - (-) | 27,11 (30,33) | 7,62 (54,05) | **1,71* (9,69)** |
|  | Active smoker | **n** | 0 | 0 | 0 | 30 | 23 | 21 |
|  |  | **Median (IQR)** | - (-) | - (-) | - (-) | 8,1 (12,64) | **2,49* (5,37)** | **0,82*$ (3,19)** |
| **Alcohol intake** | Never | **n** | 0 | 0 | 0 | 55 | 45 | 41 |
|  |  | **Median (IQR)** | - (-) | - (-) | - (-) | 4,3 (10,54) | **1,03* (8,01)** | **0,56*$ (1,8)** |
|  | Sometimes | **n** | 0 | 0 | 0 | 27 | 19 | 19 |
|  |  | **Median (IQR)** | - (-) | - (-) | - (-) | 13,97 (28,23) | **2,49* (6,21)** | **0,51*$ (3,25)** |
| **Country of origin** | Out of Spain | **n** | 2 | 2 | 2 | 42 | 32 | 31 |
|  |  | **Median (IQR)** | 85,54 (160,92) | 4,96 (9,68) | **1,48* (2,84)** | 5,71 (14,35) | **2,22* (6,59)** | **0,84*$ (3,27)** |
|  | Spain | **n** | 10 | 10 | 8 | 30 | 23 | 19 |
|  |  | **Median (IQR)** | 2 (28,05) | 0,33 (21,11) | 0,45 (2,53) | 8,73 (15) | **2,05* (5,37)** | **0,42*$ (1,25)** |
| **Comorbidities** | No | **n** | 7 | 6 | 5 | 37 | 32 | 30 |
|  |  | **Median (IQR)** | 0,9 (4,06) | 2,23 (0,28) | 0,14 (0,17) | 5,37 (9,72) | **1,19* (4,28)** | **0,38*$ (1,18)** |
|  | Yes | **n** | 1 | 1 | 0 | 24 | 15 | 16 |
|  |  | **Median (IQR)** | 2,3 (0) | 0,2 (0) | - (-) | 7,6 (20,51) | **2,1* (9,75)** | **0,67*$ (1,16)** |
| **Previous exposure to TB drugs** | No | **n** | 12 | 12 | 10 | 67 | 51 | 50 |
|  |  | **Median (IQR)** | 3,3 (46,78) | **0,33* (15,35)** | **0,45* (2,76)** | 7,41 (14,5) | **2,05* (6,69)** | **0,54*$ (1,6)** |
|  |  |  |  |  |  |  |  |  |
|  | Yes | **n** | 0 | 0 | 0 | 5 | 4 | 1 |
|  |  | **Median (IQR)** | - (-) | - (-) | - (-) | 2 (9,92) | 3,68 (4,08) | 2,37 (0) |
|  |  |  |  |  |  |  |  |  |
| **Tuberculin history** | Negative | **n** | 1 | 1 | 0 | 3 | 3 | 2 |
|  |  | **Median (IQR)** | 2,3 (0) | 0,2 (1,9) | - (-) | 0,65 (1,9) | 0,64 (0,87) | 0,09 (0,11) |
|  | Positive | **n** | 8 | 8 | 7 | 13 | 12 | 8 |
|  |  | **Median (IQR)** | 16,35 (77,4) | 8,74 (10,3) | **2* (3,1)** | 8,7 (10,3) | **3,02* (5,37)** | **0,23*$ (4,46)** |
|  | Never done | **n** | 3 | 3 | 3 | 51 | 38 | 39 |
|  |  | **Median (IQR)** | 0,35 (4,84) | 0,12 (15,08) | 0,13 (0,81) | 7,41 (15,08) | **2,08* (6,11)** | **0,7*$ (1,29)** |
| **Type of diagnosis** | Clinical | **n** | 9 | 8 | 6 | 11 | 10 | 10 |
|  |  | **Median (IQR)** | 2,3 (27,5) | 4,04 (21,95) | **0,45* (2,7)** | 1,47 (14,9) | **0,57* (2,39)** | **0,4* (0,56)** |
|  | Microbiological | **n** | 3 | 4 | 4 | 60 | 44 | 40 |
|  |  | **Median (IQR)** | 5,08 (90,65) | 0,23 (10,63) | 1,07 (5,33) | 8,1 (14,62) | **2,05* (5,88)** | **0,76*$ (3,31)** |
| **Type of TB** | Pulmonary | **n** | 7 | 6 | 5 | 43 | 34 | 30 |
|  |  | **Median (IQR)** | 1,7 (66,16) | 5,1 (34,3) | 0,6 (2,6) | 6,04 (14,49) | **1,34* (3,29)** | **0,69*$ (1,12)** |
|  | Extrapulmonary + pulmonary | **n** | 2 | 2 | 2 | 9 | 5 | 7 |
|  |  | **Median (IQR)** | 14,38 (28,05) | 3,97 (7,4) | 0,17 (0,06) | 16,4 (17,5) | 13 (45,65) | 5,82 (11,12) |
|  | Extrapulmonary | **n** | 2 | 3 | 2 | 18 | 14 | 13 |
|  |  | **Median (IQR)** | 46,65 (88,7) | 0,2 (21,1) | 5,43 (6,86) | 1,32 (8,54) | **1,05* (6,55)** | **0,25*$ (1,62)** |
|  | Disseminated | **n** | 1 | 1 | 1 | 3 | 3 | 1 |
|  |  | **Median (IQR)** | 5,08 (0) | 0,12 (0) | 0,06 (0) | 10,92 (118,26) | 6,2 (9,25) | 0,7 (0) |
| **Culture** | Negative | **n** | 8 | 7 | 5 | 4 | 3 | 3 |
|  |  | **Median (IQR)** | 2 (15,78) | 0,4 (9,6) | 0,3 (0,4) | 0,8 (3,02) | 0,34 (0,49) | 0,26 (1,64) |
|  | Positive | **n** | 4 | 5 | 5 | 64 | 50 | 47 |
|  |  | **Median (IQR)** | 35,74 (75,99) | 0,27 (21,11) | 2 (3,26) | 7,61 (14,53) | **2,30* (6,18)** | **0,7*$ (3,29)** |
| **TB-drug resistance** | Sensitive | **n** | 3 | 3 | 3 | 55 | 44 | 41 |
|  |  | **Median (IQR)** | 5,08 (66,05) | 0,27 (34,38) | 0,14 (3,34) | 7,8 (14,56) | **2* (5,63)** | **0,82*$ (2,12)** |
|  | MDR or monoresistant | **n** | 1 | 2 | 2 | 7 | 4 | 4 |
|  |  | **Median (IQR)** | 91 (0) | 10,75 (21,11) | 5,43 (6,87) | 8,76 (14,29) | 6,35 (5,66) | 0,31 (1,79) |
| **AFB** | Negative | **n** | 11 | 11 | 9 | 30 | 23 | 21 |
|  |  | **Median (IQR)** | 2,3 (28,05) | 0,27 (9,6) | **0,3* (1,86)** | 1,39 (8,54) | 0,8 (4,95) | **0,41$ (2,17)** |
|  | Positive | **n** | 1 | 1 | 1 | 34 | 25 | 25 |
|  |  | **Median (IQR)** | 64,4 (0) | 34,5 (0) | 3,4 (0) | 8,28 (12,04) | **2,05* (3,22)** | **0,42*$ (0,84)** |
| **BMI** | Underweigth | **n** | 2 | 2 | 2 | 8 | 4 | 7 |
|  |  | **Median (IQR)** | 45,6 (90,8) | 10,75 (21,11) | 4,58 (8,57) | 6,05 (52,4) | 2,23 (5,4) | **0,82* (1,27)** |
|  | Normal | **n** | 6 | 6 | 4 | 34 | 30 | 28 |
|  |  | **Median (IQR)** | 2 (3,4) | 0,33 (7,47) | 0,4 (1,13) | 6,84 (10,39) | **2,08* (6,11)** | **0,63*$ (2,19)** |
|  | Overweight | **n** | 2 | 2 | 2 | 17 | 12 | 12 |
|  |  | **Median (IQR)** | 83,12 (165,76) | 4,96 (9,68) | 1,52 (2,77) | 5,37 (11,11) | 0,54 (2,49) | 0,29 (0,95) |
| **Symptoms** | No | **n** | 2 | 2 | 2 | 10 | 8 | 6 |
|  |  | **Median (IQR)** | 2,25 (4,1) | 17,35 (34,3) | 0,45 (0,3) | 6,22 (29,14) | 2,44 (8,16) | **0,46$ (11,25)** |
|  | Yes | **n** | 10 | 10 | 8 | 63 | 48 | 45 |
|  |  | **Median (IQR)** | 3,69 (65,5) | **0,33* (9,6)** | **1,1* (3,01)** | 7,41 (13,96) | **2,05* (5,99)** | **0,56*$ (1,56)** |
| **Chest pain** | No | **n** | 10 | 9 | 7 | 37 | 33 | 29 |
|  |  | **Median (IQR)** | 25 (41) | 13 (14) | **7* (20)** | 53 (64) | 42 (98,61) | **44,7*$ (44)** |
|  | Yes | **n** | 1 | 1 | 2 | 17 | 16 | 13 |
|  |  | **Median (IQR)** | 35 (0) | 3 (0) | 2 (0) | 71 (40) | **45,3* (70,5)** | **19*$ (20)** |
| **Productive cough** | No | **n** | 8 | 7 | 8 | 25 | 20 | 20 |
|  |  | **Median (IQR)** | 25 (45,5) | 6 (45,05) | **2* (5)** | 36 (54) | 29,65 (71,56) | **21*$ (39,74)** |
|  | Yes | **n** | 4 | 4 | 3 | 29 | 30 | 22 |
|  |  | **Median (IQR)** | 39 (19,5) | 16,5 (12,5) | 12 (20) | 72 (54) | **55,5* (90)** | **45,59*$ (39,9)** |
| **Haemoptysis** | No | **n** | 12 | 11 | 10 | 45 | 40 | 36 |
|  |  | **Median (IQR)** | 32,5 (32) | **13 (19)** | **2* (5)** | 56 (63) | **43,5* (80,47)** | **28,29*$ (42,68)** |
|  | Yes | **n** | 0 | 0 | 0 | 6 | 9 | 5 |
|  |  | **Median (IQR)** | . (.) | . (.) | . (.) | 47,5 (104) | 58 (89,61) | 46,48 (15) |
| **Night Sweats** | No | **n** | 11 | 11 | 9 | 33 | 29 | 24 |
|  |  | **Median (IQR)** | 30 (41) | 9,5 (15) | **2* (5)** | 43 (59) | 41 (94,2) | **23,79*$ (38,24)** |
|  | Yes | **n** | 1 | 1 | 1 | 19 | 20 | 17 |
|  |  | **Median (IQR)** | 43 (0) | 24 (0) | 2 (0) | 72 (46) | **48,5* (66,47)** | **46,48* (40)** |
| **Shortness of breath** | No | **n** | 9 | 8 | 7 | 41 | 37 | 33 |
|  |  | **Median (IQR)** | 20 (25) | 9,5 (26,02) | 2 (5) | 68 (64) | **42* (76,94)** | **44,7*$ (42)** |
|  | Yes | **n** | 2 | 2 | 2 | 13 | 12 | 9 |
|  |  | **Median (IQR)** | 43 (16) | 11,5 (17) | 12 (20) | 50 (37) | 63,5 (88,5) | **20*$ (7)** |
| **Constitutional syndrome** | No | **n** | 6 | 6 | 5 | 29 | 25 | 20 |
|  |  | **Median (IQR)** | 32,5 (31) | 13 (43,05) | 7 (20) | 42 (54) | 33,3 (63,2) | **28,5*$ (33,23)** |
|  | Yes | **n** | 6 | 5 | 5 | 25 | 24 | 22 |
|  |  | **Median (IQR)** | 27,5 (40) | 13 (9) | 2 (0) | 79 (65) | **68,32* (87)** | **32,92*$ (40,55)** |
| **Feverish feeling** | No | **n** | 10 | 10 | 7 | 27 | 22 | 19 |
|  |  | **Median (IQR)** | 32,5 (31) | 14 (18) | **7* (20)** | 36 (52) | 37,15 (103,2) | **25*$ (10,97)** |
|  | Yes | **n** | 2 | 1 | 3 | 35 | 30 | 27 |
|  |  | **Median (IQR)** | 18,5 (33) | 3 (0) | 2 (0) | 8,7 (14,02) | **2* (4,4)** | **0,82*$ (1,13)** |
| **Lymphadenopathy** | No | **n** | 9 | 8 | 6 | 46 | 45 | 36 |
|  |  | **Median (IQR)** | 30 (15) | 9,5 (12) | **2$ (5)** | 70 (57) | **52* (84)** | **32,92*$ (37,51)** |
|  | Yes | **n** | 2 | 2 | 3 | 8 | 5 | 7 |
|  |  | **Median (IQR)** | 35,5 (67) | 25,52 (45,05) | 7 (20,39) | 29,5 (47) | 16,39 (28,04) | 9 (54,21) |

ESR

|  |  |  | **CHILDREN** | | | **ADULTS** | | |
| --- | --- | --- | --- | --- | --- | --- | --- | --- |
|  |  |  | **Baseline** | **FUM2** | **FUM6** | **Basal** | **FUM2** | **FUM6** |
| **Sex** | Men | **n** | 10 | 9 | 8 | 39 | 36 | 28 |
|  |  | **Median (IQR)** | 25 (33) | 13 (19) | **2$ (5)** | 50 (76) | 43,8 (106,57) | **26,39*$ (38,71)** |
|  | Women | **n** | 2 | 2 | 2 | 16 | 15 | 15 |
|  |  | **Median (IQR)** | 43 (16) | 13 (14) | 12 (20) | 70,5 (45,5) | **45* (30,7)** | **33,83* (40,56)** |
| **Age** | <18 | **n** | 12 | 11 | 10 | 0 | 0 | 0 |
|  |  | **Median (IQR)** | 32,5 (32) | 13 (19) | **2* (5)** | - (-) | - (-) | - (-) |
|  | 18 - 40 | **n** | 0 | 0 | 0 | 19 | 21 | 13 |
|  |  | **Median (IQR)** | . (.) | . (.) | . (.) | 47 (75) | **33,30* (67,63)** | **28*$ (25)** |
|  | 41    – 60 | **n** | 0 | 0 | 0 | 28 | 28 | 25 |
|  |  | **Median (IQR)** | . (.) | . (.) | . (.) | 70,5 (66,5) | 57 (92) | **32*$ (42)** |
|  | > 60 | **n** | 0 | 0 | 0 | 8 | 5 | 5 |
|  |  | **Median (IQR)** | . (.) | . (.) | . (.) | 42,5 (45,5) | 45 (10) | 39 (35,7) |
| **Smoking habit** | Never smoked | **n** | 0 | 0 | 0 | 35 | 33 | 28 |
|  |  | **Median (IQR)** | . (.) | . (.) | . (.) | 42 (60) | **20* (46)** | **16,23*$ (45,46)** |
|  | Ex-smoker | **n** | 0 | 0 | 0 | 5 | 4 | 5 |
|  |  | **Median (IQR)** | . (.) | . (.) | . (.) | 48 (31) | 22 (38,5) | 20 (4) |
|  | Active smoker | **n** | 0 | 0 | 0 | 27 | 25 | 20 |
|  |  | **Median (IQR)** | . (.) | . (.) | . (.) | 72 (84) | **75* (79)** | **32,91*$ (47,5)** |
| **Alcohol intake** | No | **n** | 0 | 0 | 0 | 45 | 40 | 38 |
|  |  | **Median (IQR)** | . (.) | . (.) | . (.) | 53 (52) | **27* (53,1)** | **21,5*$ (47)** |
|  | Yes | **n** | 0 | 0 | 0 | 20 | 19 | 15 |
|  |  | **Median (IQR)** | . (.) | . (.) | . (.) | 59 (61) | 79 (81,7) | **32*$ (32)** |
| **Country of origin** | Out of Spain | **n** | 3 | 2 | 2 | 28 | 28 | 25 |
|  |  | **Median (IQR)** | 35 (94) | 9 (12) | 4,5 (5) | 55,5 (59,5) | **38,22* (68,64)** | **32* (40)** |
|  | Spain | **n** | 9 | 9 | 8 | 26 | 22 | 17 |
|  |  | **Median (IQR)** | 30 (23) | 13 (18) | **2* (12,5)** | 68,5 (64) | 54,5 (85) | **32*$ (33)** |
| **Comorbidities** | Yes | **n** | 8 | 7 | 5 | 28 | 29 | 28 |
|  |  | **Median (IQR)** | 32,5 (40) | 15 (42,04) | **7* (20)** | 71,5 (78) | **41* (84)** | **36,42*$ (40,73)** |
|  | No | **n** | 1 | 1 | 0 | 19 | 15 | 13 |
|  |  | **Median (IQR)** | 3 (0) | 6 (0) | . (.) | 68 (54) | 75 (76) | **20*$ (32,7)** |
| **Previous exposure to TB drugs** | No | **n** | 12 | 11 | 10 | 51 | 46 | 43 |
|  |  | **Median (IQR)** | 32,5 (32) | 13 (19) | **2* (5)** | 68 (67) | **52,5* (85)** | **32*$ (42)** |
|  | Yes | **n** | 0 | 0 | 0 | 4 | 4 | 0 |
|  |  | **Median (IQR)** | . (.) | . (.) | . (.) | 26 (51,5) | 4 (22,5) | . (.) |
| **Tuberculin history** | Negative | **n** | 2 | 1 | 0 | 3 | 3 | 2 |
|  |  | **Median (IQR)** | 6,5 (7) | 6 (0) | . (.) | 9 (116) | 11,8 (115) | 39,01 (63,98) |
|  | Positive | **n** | 7 | 7 | 7 | 9 | 10 | 7 |
|  |  | **Median (IQR)** | 30 (15) | 6 (21) | 2 (0) | 27 (50) | 14,93 (40) | 12 (47,21) |
|  | Never done | **n** | 3 | 3 | 3 | 39 | 34 | 32 |
|  |  | **Median (IQR)** | 69 (53) | 20 (33,05) | 22 (15,39) | 71 (61) | **61* (79)** | **32*$ (37,51)** |
| **Type of diagnosis** | Clinical | **n** | 9 | 8 | 6 | 9 | 9 | 9 |
|  |  | **Median (IQR)** | 30 (15) | 9,5 (16,5) | **2* (0)** | 78 (65) | **39,84* (38)** | **28*$ (42)** |
|  | Microbiological | **n** | 3 | 3 | 4 | 46 | 42 | 34 |
|  |  | **Median (IQR)** | 69 (102) | 15 (45,05) | 7 (10,2) | 54,5 (55) | 45,3 (95,2) | **32* (37,48)** |
| **Type of TB** | Pulmonary | **n** | 8 | 7 | 5 | 34 | 33 | 25 |
|  |  | **Median (IQR)** | 32,5 (19) | 13 (19) | **2* (0)** | 68,5 (78) | **52* (87)** | **44,7*$ (39)** |
|  | ExtraP + pulmonary | **n** | 1 | 1 | 2 | 3 | 3 | 4 |
|  |  | **Median (IQR)** | 69 (0) | 48,05 (0) | 12,2 (20,39) | 72 (37) | 107 (42,37) | 20,5 (11,89) |
|  | Extrapulmonary | **n** | 2 | 2 | 2 | 15 | 12 | 13 |
|  |  | **Median (IQR)** | 2,5 (1) | 4,5 (3) | 4,5 (5) | 47 (51) | 34,96 (39,97) | **20* (45,94)** |
|  | Disseminated | **n** | 1 | 1 | 1 | 3 | 3 | 1 |
|  |  | **Median (IQR)** | 104 (0) | 15 (0) | 7 (0) | 32 (71) | 45 (111) | 72 (0) |
| **Culture** | Negative | **n** | 8 | 7 | 5 | 4 | 3 | 3 |
|  |  | **Median (IQR)** | 25 (20) | 6 (15) | 2 (0) | 63 (29) | 36,61 (17,84) | 52,96 (52) |
|  | Positive | **n** | 4 | 4 | 5 | 48 | 45 | 39 |
|  |  | **Median (IQR)** | 56 (64) | 19,5 (27,02) | 7 (5) | 68,5 (72) | 53 (88) | **32*$ (42)** |
| **TB-drug resistance** | Sensitive | **n** | 3 | 3 | 3 | 40 | 40 | 34 |
|  |  | **Median (IQR)** | 69 (61) | 24 (33,05) | 7 (20,39) | 63 (61,5) | 48,8 (81,97) | **32,92*$ (41)** |
|  | MDR or MonoR | **n** | 1 | 1 | 2 | 7 | 4 | 4 |
|  |  | **Median (IQR)** | 2 (0) | 3 (0) | 4,5 (5) | 68 (109) | 120 (57,75) | 18,5 (38,22) |
| **AFB** | Negative | **n** | 11 | 10 | 9 | 25 | 21 | 18 |
|  |  | **Median (IQR)** | 30 (41) | 9,5 (15) | **2* (5)** | 47 (51) | **26* (34)** | **24,39*$ (39,94)** |
|  | Positive | **n** | 1 | 1 | 1 | 27 | 26 | 23 |
|  |  | **Median (IQR)** | 43 (0) | 24 (0) | 2 (0) | 85 (70) | 89,33 (75) | **45*$ (42)** |
| **BMI** | Underweigth | **n** | 2 | 1 | 2 | 6 | 4 | 5 |
|  |  | **Median (IQR)** | 11 (18) | 5 (0) | 2 (0) | 114 (24) | 107,47 (58,53) | **70* (48)** |
|  | Normal | **n** | 6 | 6 | 4 | 28 | 28 | 24 |
|  |  | **Median (IQR)** | 25 (25) | 9,5 (42,05) | 4,5 (12,7) | 70 (63) | 57,5 (64,68) | **29,89*$ (30,24)** |
|  | Overweight | **n** | 2 | 2 | 2 | 13 | 11 | 13 |
|  |  | **Median (IQR)** | 43 (16) | 11,5 (17) | 12 (20) | 55 (42) | 39,84 (85) | 28 (40,97) |
| **Symptoms** | No | **n** | 3 | 2 | 2 | 9 | 7 | 5 |
|  |  | **Median (IQR)** | 20 (20) | 36 (62) | 2 (0) | 32 (29) | 19 (36) | 32 (36,48) |
|  | Yes | **n** | 9 | 9 | 8 | 46 | 44 | 38 |
|  |  | **Median (IQR)** | 35 (31) | **13* (14)** | **4,5* (12,5)** | 71 (62) | **52,5* (84,5)** | **30*$ (42)** |
| **Chest pain** | No | **n** | 10 | 9 | 7 | 37 | 33 | 29 |
|  |  | **Median (IQR)** | 25 (41) | 13 (14) | **7* (20)** | 53 (64) | 42 (98,61) | **44,7*$ (44)** |
|  | Yes | **n** | 1 | 1 | 2 | 17 | 16 | 13 |
|  |  | **Median (IQR)** | 35 (0) | 3 (0) | 2 (0) | 71 (40) | **45,3* (70,5)** | **19*$ (20)** |
| **Productive cough** | No | **n** | 8 | 7 | 8 | 25 | 20 | 20 |
|  |  | **Median (IQR)** | 25 (45,5) | 6 (45,05) | **2* (5)** | 36 (54) | 29,65 (71,56) | **21*$ (39,74)** |
|  | Yes | **n** | 4 | 4 | 3 | 29 | 30 | 22 |
|  |  | **Median (IQR)** | 39 (19,5) | 16,5 (12,5) | 12 (20) | 72 (54) | **55,5* (90)** | **45,59*$ (39,9)** |
| **Haemoptysis** | No | **n** | 12 | 11 | 10 | 45 | 40 | 36 |
|  |  | **Median (IQR)** | 32,5 (32) | 13 (19) | **2* (5)** | 56 (63) | **43,5* (80,47)** | **28,29*$ (42,68)** |
|  | Yes | **n** | 0 | 0 | 0 | 6 | 9 | 5 |
|  |  | **Median (IQR)** | . (.) | . (.) | . (.) | 47,5 (104) | 58 (89,61) | 46,48 (15) |
| **Night Sweats** | No | **n** | 11 | 10 | 9 | 33 | 29 | 24 |
|  |  | **Median (IQR)** | 30 (41) | 9,5 (15) | **2* (5)** | 43 (59) | 41 (94,2) | **23,79*$ (38,24)** |
|  | Yes | **n** | 1 | 1 | 1 | 19 | 20 | 17 |
|  |  | **Median (IQR)** | 43 (0) | 24 (0) | 2 (0) | 72 (46) | **48,5* (66,47)** | **46,48* (40)** |
| **Shortness of breath** | No | **n** | 9 | 8 | 7 | 41 | 37 | 33 |
|  |  | **Median (IQR)** | 20 (25) | 9,5 (26,02) | 2 (5) | 68 (64) | **42* (76,94)** | **44,7*$ (42)** |
|  | Yes | **n** | 2 | 2 | 2 | 13 | 12 | 9 |
|  |  | **Median (IQR)** | 43 (16) | 11,5 (17) | 12 (20) | 50 (37) | 63,5 (88,5) | **20*$ (7)** |
| **Constitutional syndrome** | No | **n** | 6 | 6 | 5 | 29 | 25 | 20 |
|  |  | **Median (IQR)** | 32,5 (31) | 13 (43,05) | 7 (20) | 42 (54) | 33,3 (63,2) | **28,5*$ (33,23)** |
|  | Yes | **n** | 6 | 5 | 5 | 25 | 24 | 22 |
|  |  | **Median (IQR)** | 27,5 (40) | 13 (9) | 2 (0) | 79 (65) | **68,32* (87)** | **32,92*$ (40,55)** |
| **Feverish feeling** | No | **n** | 10 | 10 | 7 | 27 | 22 | 19 |
|  |  | **Median (IQR)** | 32,5 (31) | 14 (18) | **7* (20)** | 36 (52) | 37,15 (103,2) | **25*$ (10,97)** |
|  | Yes | **n** | 2 | 1 | 3 | 27 | 28 | 23 |
|  |  | **Median (IQR)** | 18,5 (33) | 3 (0) | 2 (0) | 79 (64) | **55,5* (73,47)** | **45*$ (40,55)** |
| **Lymphadenopathy** | No | **n** | 9 | 8 | 6 | 46 | 45 | 36 |
|  |  | **Median (IQR)** | 30 (15) | 9,5 (12) | **2$ (5)** | 70 (57) | **52* (84)** | **32,92*$ (37,51)** |
|  | Yes | **n** | 2 | 2 | 3 | 8 | 5 | 7 |
|  |  | **Median (IQR)** | 35,5 (67) | 25,52 (45,05) | 7 (20,39) | 29,5 (47) | 16,39 (28,04) | 9 (54,21) |

NLR

|  |  |  | **CHILDREN** | | | **ADULTS** | | |
| --- | --- | --- | --- | --- | --- | --- | --- | --- |
|  |  |  | **Baseline** | **FUM2** | **FUM6** | **Baseline** | **FUM2** | **FUM6** |
| **Sex** | Men | **n** | 10 | 10 | 8 | 56 | 48 | 39 |
|  |  | **Median (IQR)** | 2,26 (3) | 1,58 (1,74) | **1,02* (1,46)** | 3,43 (2,56) | **2,79* (2,28)** | **2,29*$ (0,8)** |
|  | Women | **n** | 3 | 3 | 2 | 21 | 20 | 19 |
|  |  | **Median (IQR)** | 2,34 (1,7) | 1,46 (0,61) | 0,77 (0,3) | 3,21 (1,23) | 2,59 (0,84) | **1,91*$ (0,91)** |
| **Age** | <18 | **n** | 13 | 13 | 10 | 0 | 0 | 0 |
|  |  | **Median (IQR)** | 2,34 (1,48) | **1,46* (1,97)** | **0,93* (0,36)** | - (-) | - (-) | - (-) |
|  | 18 - 40 | **n** | 0 | 0 | 0 | 30 | 26 | 20 |
|  |  | **Median (IQR)** | . (.) | . (.) | . (.) | 2,98 (1,92) | **2,26* (1,76)** | **1,92*$ (0,98)** |
|  | 41    – 60 | **n** | 0 | 0 | 0 | 33 | 30 | 28 |
|  |  | **Median (IQR)** | . (.) | . (.) | . (.) | 3,91 (2,72) | **2,80* (1,22)** | **2,23*$ (0,82)** |
|  | > 60 | **n** | 0 | 0 | 0 | 15 | 13 | 11 |
|  |  | **Median (IQR)** | . (.) | . (.) | . (.) | 3,21 (1,67) | 2,93 (2,37) | **2,01* (2,15)** |
| **Smoking habit** | Never smoked | **n** | 0 | 0 | 0 | 47 | 44 | 33 |
|  |  | **Median (IQR)** | . (.) | . (.) | . (.) | 2,86 (2,9) | **2,20* (1,76)** | **1,85*$ (1,31)** |
|  | Ex-smoker | **n** | 0 | 0 | 0 | 10 | 9 | 8 |
|  |  | **Median (IQR)** | . (.) | . (.) | . (.) | 3,69 (3,64) | 3,34 (2,25) | **2,35*$ (2,02)** |
|  | Active smoker | **n** | 0 | 0 | 0 | 33 | 28 | 27 |
|  |  | **Median (IQR)** | . (.) | . (.) | . (.) | 3,38 (2,09) | **2,71* (1,08)** | **2,05*$ (0,85)** |
| **Alcohol intake** | No | **n** | 0 | 0 | 0 | 58 | 53 | 48 |
|  |  | **Median (IQR)** | . (.) | . (.) | . (.) | 2,86 (2,58) | **2,23* (1,34)** | **1,88*$ (1,18)** |
|  | Yes | **n** | 0 | 0 | 0 | 28 | 24 | 20 |
|  |  | **Median (IQR)** | . (.) | . (.) | . (.) | 3,96 (3,84) | **2,97* (2,29)** | **2,43*$ (1,34)** |
| **Country of origin** | Out of Spain | **n** | 3 | 2 | 2 | 44 | 39 | 33 |
|  |  | **Median (IQR)** | 2,58 (4,68) | 1,89 (1,63) | 2,48 (2,72) | 3,1 (2,51) | **2,62* (1,65)** | **1,94*$ (0,94)** |
|  | Spain | **n** | 10 | 11 | 8 | 33 | 29 | 25 |
|  |  | **Median (IQR)** | 2,11 (1,54) | **1,46* (1,24)** | **0,92*$ (0,29)** | 3,64 (2,37) | **2,71* (2,25)** | **2,35*$ (0,89)** |
| **Comorbidities** | Yes | **n** | 1 | 1 | 0 | 28 | 23 | 22 |
|  |  | **Median (IQR)** | 5,03 (0) | 1,28 (0) | . (.) | 3,08 (2,35) | 2,76 (1,34) | **2,40*$ (0,8)** |
|  | No | **n** | 8 | 7 | 5 | 38 | 34 | 32 |
|  |  | **Median (IQR)** | 1,62 (0,99) | **0,97* (0,58)** | **0,92* (0,07)** | 3,56 (2,49) | **2,74* (1,27)** | **1,88*$ (0,86)** |
| **Previous exposure to TB drugs** | Yes | **n** | 0 | 0 | 0 | 7 | 5 | 2 |
|  |  | **Median (IQR)** | . (.) | . (.) | . (.) | 1,94 (1) | 1,82 (0,85) | 2,05 (1,67) |
|  | No | **n** | 13 | 13 | 10 | 70 | 63 | 57 |
|  |  | **Median (IQR)** | 2,34 (1,48) | **1,46* (1,2)** | **0,93* (0,36)** | 3,58 (2,49) | **2,76* (1,39)** | **2,01*$ (0,95)** |
| **Tuberculin history** | Negative | **n** | 2 | 1 | 0 | 3 | 3 | 2 |
|  |  | **Median (IQR)** | 3,48 (3,09) | 1,28 (0) | . (.) | 2,84 (1,98) | 2,19 (2,25) | 1,5 (0,9) |
|  | Positive | **n** | 8 | 9 | 7 | 14 | 13 | 9 |
|  |  | **Median (IQR)** | 2,51 (1,99) | 1,88 (1,24) | **0,92* (2,77)** | 2,33 (1,6) | 2,04 (1,46) | 1,6 (0,89) |
|  | Never done | **n** | 3 | 3 | 3 | 55 | 49 | 46 |
|  |  | **Median (IQR)** | 1,32 (1,61) | 0,92 (0,19) | 0,93 (0,2) | 3,74 (3,22) | **2,71* (2,21)** | **2,22*$ (0,84)** |
| **Type of diagnosis** | Clinical | **n** | 10 | 9 | 6 | 11 | 10 | 10 |
|  |  | **Median (IQR)** | 2,14 (3) | 1,46 (0,91) | **0,92* (0,51)** | 3,11 (3,82) | 2,92 (2,18) | **2,17*$ (0,9)** |
|  | Microbiological | **n** | 3 | 4 | 4 | 65 | 57 | 48 |
|  |  | **Median (IQR)** | 2,58 (1,54) | 1,62 (2,01) | 1,02 (1,38) | 3,38 (2,4) | **2,64* (1,29)** | **2,00*$ (0,97)** |
| **Type of TB** | Pulmonary | **n** | 8 | 7 | 5 | 48 | 43 | 35 |
|  |  | **Median (IQR)** | 1,9 (2,18) | 1,46 (1,82) | **0,92* (0,36)** | 3,5 (2,55) | **2,66* (1,18)** | **2,00*$ (0,83)** |
|  | ExtraP + pulmonary | **n** | 2 | 2 | 2 | 10 | 8 | 8 |
|  |  | **Median (IQR)** | 2 (1,35) | 1,21 (0,57) | 0,78 (0,3) | 3,84 (2,8) | 2,97 (1,91) | **2,13$ (1,59)** |
|  | Extrapulmonary | **n** | 2 | 3 | 2 | 17 | 15 | 14 |
|  |  | **Median (IQR)** | 3,94 (2,17) | 2,17 (2,58) | 2,21 (2,56) | 2,59 (1,03) | 2,18 (1,84) | **1,96*$ (1,22)** |
|  | Disseminated | **n** | 1 | 1 | 1 | 3 | 3 | 2 |
|  |  | **Median (IQR)** | 2,58 (0) | 1,08 (0) | 1,12 (0) | 4,14 (1,83) | 5,59 (3,92) | 2,75 (2,27) |
| **Culture** | Negative | **n** | 9 | 8 | 5 | 4 | 3 | 4 |
|  |  | **Median (IQR)** | 1,93 (1,3) | **1,37* (0,76)** | **0,86* (0,21)** | 2,03 (3,16) | 3,06 (2,92) | 1,82 (2,02) |
|  | Positive | **n** | 4 | 5 | 5 | 69 | 63 | 54 |
|  |  | **Median (IQR)** | 2,72 (1,66) | 2,17 (2,78) | 1,12 (0,3) | 3,48 (2,5) | **2,71* (1,42)** | **2,03*$ (0,96)** |
| **TB-drug resistance** | Sensitive | **n** | 3 | 3 | 3 | 60 | 56 | 48 |
|  |  | **Median (IQR)** | 2,58 (3,05) | 1,08 (4,04) | 1,12 (0,3) | 3,48 (2,46) | **2,63* (1,37)** | **2,00*$ (0,97)** |
|  | MDR or monoR | **n** | 1 | 2 | 2 | 7 | 5 | 4 |
|  |  | **Median (IQR)** | 2,86 (0) | 3,01 (1,7) | 2,21 (2,56) | 3,99 (4,04) | 2,93 (0,36) | 2,49 (0,42) |
| **AFB** | Negative | **n** | 12 | 12 | 9 | 36 | 32 | 27 |
|  |  | **Median (IQR)** | 2,14 (1,42) | **1,37* (1,08)** | **0,92* (0,26)** | 2,65 (2,01) | 2,35 (1,63) | **2,18* (0,84)** |
|  | Positive | **n** | 1 | 1 | 1 | 33 | 29 | 27 |
|  |  | **Median (IQR)** | 4,37 (0) | 4,96 (0) | 1,22 (0) | 3,71 (2,31) | **2,79* (0,82)** | **1,95*$ (0,84)** |
| **BMI** | Underweigth | **n** | 2 | 2 | 2 | 9 | 7 | 8 |
|  |  | **Median (IQR)** | 2,12 (1,48) | 1,57 (1,2) | 0,891 (0,06) | 5,12 (8,11) | **2,66* (1,05)** | **1,94*$ (0,93)** |
|  | Normal | **n** | 7 | 7 | 4 | 35 | 34 | 32 |
|  |  | **Median (IQR)** | 1,93 (1,35) | **1,46* (0,95)** | 0,82 (1,54) | 3,35 (2,32) | **2,78* (1,33)** | **2,03*$ (0,71)** |
|  | Overweight | **n** | 2 | 2 | 2 | 19 | 16 | 16 |
|  |  | **Median (IQR)** | 3,79 (6,63) | 1,8 (1,82) | 2,38 (2,92) | 2,81 (2,25) | 2,67 (1,86) | **1,96* (1,46)** |
| **Symptoms** | No | **n** | 3 | 2 | 2 | 10 | 9 | 5 |
|  |  | **Median (IQR)** | 1,87 (0,56) | 1,42 (0,91) | 0,79 (0,45) | 4,04 (1,27) | 3,19 (1,49) | 2,61 (1,81) |
|  | Yes | **n** | 10 | 11 | 8 | 68 | 60 | 54 |
|  |  | **Median (IQR)** | 2,63 (3,05) | **1,46* (1,79)** | **1,02 *(1,43)** | 3,3 (2,6) | **2,59* (1,4)** | **1,97*$ (0,9)** |
| **Chest pain** | No | **n** | 10 | 10 | 7 | 51 | 44 | 37 |
|  |  | **Median (IQR)** | 1,9 (1,26) | **1,18* (0,95)** | **0,92* (0,26)** | 3,71 (2,73) | **2,64* (1,2)** | **1,94*$ (0,88)** |
|  | Yes | **n** | 2 | 2 | 2 | 24 | 22 | 20 |
|  |  | **Median (IQR)** | 4,64 (3,94) | 2,1 (1,21) | 2,24 (3,22) | 3,04 (1,8) | 2,78 (2,24) | **2,36*$ (0,75)** |
| **Productive cough** | No | **n** | 9 | 9 | 8 | 37 | 33 | 28 |
|  |  | **Median (IQR)** | 2,58 (0,99) | **1,49* (1,09)** | **0,93* (1,52)** | 3,09 (1,94) | 2,62 (2,43) | **1,96*$ (1,06)** |
|  | Yes | **n** | 4 | 4 | 2 | 40 | 35 | 30 |
|  |  | **Median (IQR)** | 1,66 (2,47) | 1,17 (2,48) | 1,07 (0,3) | 3,5 (2,93) | **2,76* (1,16)** | **2,28*$ (0,96)** |
| **Haemoptysis** | No | **n** | 13 | 13 | 10 | 61 | 55 | 49 |
|  |  | **Median (IQR)** | 2,34 (1,48) | **1,46* (1,2)** | **0,93* (0,36)** | 3,38 (2,1) | **2,79* (2,29)** | **2,00*$ (0,97)** |
|  | Yes | **n** | 0 | 0 | 0 | 12 | 11 | 7 |
|  |  | **Median (IQR)** | . (.) | . (.) | . (.) | 3,12 (3,75) | **2,23* (1,19)** | **2,44* (1,09)** |
| **Night Sweats** | No | **n** | 12 | 12 | 9 | 50 | 43 | 34 |
|  |  | **Median (IQR)** | 2,14 (1,42) | **1,37* (1,08)** | **0,92* (0,26)** | 3,1 (1,93) | **2,71* (1,29)** | **2,11*$ (0,6)** |
|  | Yes | **n** | 1 | 1 | 1 | 24 | 23 | 22 |
|  |  | **Median (IQR)** | 4,37 (0) | 4,96 (0) | 1,22 (0) | 3,54 (3,31) | **2,57* (2,68)** | **1,96*$ (1,29)** |
| **Shortness of breath** | No | **n** | 10 | 10 | 7 | 60 | 53 | 45 |
|  |  | **Median (IQR)** | 2,14 (1,3) | **1,37* (0,91)** | **0,92* (0,41)** | 3,43 (2,77) | **2,71* (2,23)** | **2,05*$ (1,05)** |
|  | Yes | **n** | 2 | 2 | 2 | 16 | 14 | 13 |
|  |  | **Median (IQR)** | 3,79 (5,63) | 1,8 (1,82) | 2,38 (2,92) | 3,42 (2,75) | **2,56* (1,3)** | **2,00*$ (0,63)** |
| **Constitutional syndrome** | No | **n** | 6 | 6 | 5 | 38 | 33 | 25 |
|  |  | **Median (IQR)** | 1,62 (0,61) | 1,22 (0,95) | 0,92 (0,07) | 2,89 (1,98) | **2,51* (1,37)** | **2,29* (0,68)** |
|  | Yes | **n** | 7 | 7 | 5 | 36 | 33 | 33 |
|  |  | **Median (IQR)** | 2,86 (2,44) | **1,49* (1,63)** | **1,12* (0,3)** | 3,87 (2,64) | **2,66* (2,14)** | **1,95*$ (1,02)** |
| **Feverish feeling** | No | **n** | 10 | 10 | 7 | 38 | 32 | 25 |
|  |  | **Median (IQR)** | 1,9 (1,26) | 1,18 (0,95) | **0,93* (0,36)** | 3,02 (1,91) | **2,82 (2,32)** | **2,29*$ (0,8)** |
|  | Yes | **n** | 3 | 3 | 3 | 38 | 35 | 33 |
|  |  | **Median (IQR)** | 2,86 (3,94) | 2,17 (1,21) | 0,92 (3,22) | 3,5 (2,29) | **2,64* (1,21)** | **1,95*$ (0,88)** |
| **Lymphadenopathy** | No | **n** | 10 | 9 | 6 | 65 | 58 | 51 |
|  |  | **Median (IQR)** | 2,14 (1,3) | **1,28* (0,52)** | **0,89* (0,41)** | 3,64 (2,38) | **2,80* (2,07)** | **2,18*$ (0,84)** |
|  | Yes | **n** | 2 | 3 | 3 | 12 | 10 | 8 |
|  |  | **Median (IQR)** | 2,09 (1,54) | 2,17 (2,94) | 0,93 (2,56) | 1,77 (1,6) | 1,92 (1,28) | 1,37 (1,11) |

MLR

|  |  |  | **CHILDREN** | | | **ADULTS** | | |
| --- | --- | --- | --- | --- | --- | --- | --- | --- |
|  |  |  | **Baseline** | **FUM2** | **FUM6** | **Baseline** | **FUM2** | **FUM6** |
| **Sex** | Men | **n** | 9 | 9 | 7 | 55 | 49 | 40 |
|  |  | **Median (IQR)** | 0,3(0,2 | 0,2(0,1) | **0,2* (0,1)** | 0,5 (0,5) | 0,4-(0,2) | **0,3*$ (0,2)** |
|  | Women | **n** | 3 | 3 | 2 | 23 | 21 | 20 |
|  |  | **Median (IQR)** | 0,4(0,4) | 0,2(0) | 0,1(0,1) | 0,3(0,3) | 0,3(0,1) | **0,3*$ (0,1)** |
| **Age** | <18 | **n** | 12 | 12 | 9 | 0 | 0 | 0 |
|  |  | **Median (IQR)** | 0,3(0,2) | 0,2(0,05) | 0,2 (0) | - (-) | - (-) | - (-) |
|  | 18 - 40 | **n** | 0 | 0 | 0 | 30 | 27 | 21 |
|  |  | **Median (IQR)** | . (.) | . (.) | . (.) | 0,4(0,3) | 0,4(0,2) | **0,3*$ (0,1)** |
|  | 41    – 60 | **n** | 0 | 0 | 0 | 33 | 30 | 28 |
|  |  | **Median (IQR)** | . (.) | . (.) | . (.) | 0,5(0,5) | 0,4 (0,2) | **0,3*$ (0,2)** |
|  | > 60 | **n** | 0 | 0 | 0 | 13 | 13 | 11 |
|  |  | **Median (IQR)** | . (.) | . (.) | . (.) | 0,4 (0,5) | 0,4(0,5) | 0,4 (0,3) |
| **Smoking habit** | Never smoked | **n** | 0 | 0 | 0 | 35 | 32 | 25 |
|  |  | **Median (IQR)** | . (.) | . (.) | . (.) | 0,4(0,3) | 0,3 (0,1) | **0,3*$ (0,1)** |
|  | Ex-smoker | **n** | 0 | 0 | 0 | 10 | 9 | 8 |
|  |  | **Median (IQR)** | . (.) | . (.) | . (.) | 0,5(0,5) | 0,5(0,3) | **0,4* (0,2)** |
|  | Active smoker | **n** | 0 | 0 | 0 | 32 | 28 | 26 |
|  |  | **Median (IQR)** | . (.) | . (.) | . (.) | 0,5(0,3) | 0,4 (0,2) | **0,3*$ (0,1)** |
| **Alcohol intake** | No | **n** | 0 | 0 | 0 | 45 | 41 | 39 |
|  |  | **Median (IQR)** | . (.) | . (.) | . (.) | 0,4(0,3) | 0,4 (0,1) | **0,3*$ (0,2)** |
|  | Yes | **n** | 0 | 0 | 0 | 28 | 24 | 20 |
|  |  | **Median (IQR)** | . (.) | . (.) | . (.) | 0,5(0,6) | 0,4 (0,3) | **0,4*$ (0,2)** |
| **Country of origin** | Out of Spain | **n** | 3 | 2 | 2 | 44 | 40 | 34 |
|  |  | **Median (IQR)** | 0,4(0,4) | 0,2(0) | 0,3(0,2) | 0,3(0,2) | 0,3 (0,1) | **0,3*$ (0,1)** |
|  | Spain | **n** | 9 | 10 | 7 | 34 | 30 | 26 |
|  |  | **Median (IQR)** | 0,3(0,1) | 0,2(0,1) | **0,2$ (0,1)** | 0,5(0,4) | 0,4 (0,4) | **0,4*$ (0,2)** |
| **Comorbidities** | Yes | **n** | 1 | 1 | 0 | 29 | 24 | 23 |
|  |  | **Median (IQR)** | 0,4(0) | 0,2(0) | . (.) | 0,4 (0,3) | 0,3 (0,2) | **0,3*$ (0,2)** |
|  | No | **n** | 7 | 6 | 4 | 37 | 34 | 32 |
|  |  | **Median (IQR)** | 0,2(0,2) | 0,2 (0) | 0,2 (0) | 0,5 (0,4) | 0,4 (0,2) | **0,3* (0,2)** |
| **Previous exposure to TB drugs** | Yes | **n** | 0 | 0 | 0 | 7 | 6 | 2 |
|  |  | **Median (IQR)** | . (.) | . (.) | . (.) | 0,3 (0,2) | 0,3 (0,1) | 0,2 (0,4) |
|  | No | **n** | 12 | 12 | 9 | 70 | 63 | 58 |
|  |  | **Median (IQR)** | 0,3(0,2) | 0,2 (0,5) | 0,2 (0) | 0,5(0,4) | **0,4* (0,2)** | **0,3*$ (0,2)** |
| **Tuberculin history** | Negative | **n** | 2 | 1 | 0 | 3 | 3 | 2 |
|  |  | **Median (IQR)** | 0,3(0,2) | 0,2(0) | . (.) | 0,5(0,1) | 0,4(0,2) | 0,3(0,1) |
|  | Positive | **n** | 8 | 9 | 7 | 14 | 13 | 9 |
|  |  | **Median (IQR)** | 0,3(0,15) | 0,2(0,1) | **0,2* (0,2)** | 0,3(0,2) | 0,3(0,2) | 0,3(0,1) |
|  | Never done | **n** | 2 | 2 | 2 | 56 | 50 | 47 |
|  |  | **Median (IQR)** | 0,2(0,3) | 0,2(0) | 0,2(0) | 0,5(0,5) | 0,4(0,2) | **0,3*$ (0,2)** |
| **Type of diagnosis** | Clinical | **n** | 10 | 9 | 6 | 12 | 10 | 10 |
|  |  | **Median (IQR)** | 0,3(0,2) | 0,2(0) | 0,2 (0) | 0,3(0,5) | 0,4(0,2) | **0,3*$ (0,2)** |
|  | Microbiological | **n** | 2 | 3 | 3 | 66 | 60 | 50 |
|  |  | **Median (IQR)** | 0,3(0,2) | 0,2(0) | 0,2(0,2) | 0,5(0,3) | 0,4 (0,2) | **0,3*$ (0,1)** |
| **Type of TB** | Pulmonary | **n** | 8 | 7 | 5 | 47 | 42 | 34 |
|  |  | **Median (IQR)** | 0,25(0,2) | 0,2(0,1) | 0,2 (0) | 0,5(0,5) | **0,3* (0,2)** | **0,3*$ (0,1)** |
|  | ExtraP + pulmonary | **n** | 1 | 1 | 1 | 10 | 8 | 8 |
|  |  | **Median (IQR)** | 0,4(0) | 0,2(0) | 0,1(0) | 0,5(0,4) | 0,4(0,3) | **0,2*$ (0,2)** |
|  | Extrapulmonary | **n** | 2 | 3 | 2 | 18 | 17 | **16** |
|  |  | **Median (IQR)** | 0,3(0,2) | 0,2(0,1) | 0,2(0,2) | 0,3(0,1) | 0,4(0,1) | **0,3$(0,1)** |
|  | Disseminated | **n** | 1 | 1 | 1 | 3 | 3 | 2 |
|  |  | **Median (IQR)** | 0,4(0) | 0,2(0) | 0,2(0) | 0,5(0,1) | 0,6(0,4) | 0,4(0) |
| **Culture** | Negative | **n** | 9 | 8 | 5 | 4 | 4 | 4 |
|  |  | **Median (IQR)** | 0,3(0,1) | 0,2 (0) | 0,2 (0) | 0,3(0,2) | 0,4 (0,1) | 0,2 (0,3) |
|  | Positive | **n** | 3 | 4 | 4 | 70 | 63 | 55 |
|  |  | **Median (IQR)** | 0,3(0,2) | 0,25(0,05) | 0,2(0,1) | 0,4 (0,4) | **0,4* (0,1)** | **0,3*$ (0,2)** |
| **TB-drug resistance** | Sensitive | **n** | 2 | 2 | 2 | 60 | 55 | 48 |
|  |  | **Median (IQR)** | 0,3(0,1) | 0,3(0,2) | 0,2(0) | 0,5(0,3) | **0,3* (0,2)** | **0,3*$ (0,22)** |
|  | MDR or monoR | **n** | 1 | 2 | 2 | 8 | 6 | 5 |
|  |  | **Median (IQR)** | 0,2(0) | 0,25(0,1) | 0,2(0,2) | 0,4(0,6) | 0,3(1) | 0,4(0,3) |
| **AFB** | Negative | **n** | 11 | 11 | 8 | 35 | 32 | 26 |
|  |  | **Median (IQR)** | 0,3(0,2) | **0,2* (0)** | 0,2 (0,1) | 0,4 (0,4) | 0,4 (0,1) | **0,3*$ (0,2)** |
|  | Positive | **n** | 1 | 1 | 1 | 35 | 31 | 29 |
|  |  | **Median (IQR)** | 0,3(0) | 0,4(0) | 0,2(0) | 0,5 (0,4) | 0,3 (0,3) | **0,3*$ (0,2)** |
| **BMI** | Underweigth | **n** | 2 | 2 | 2 | 9 | 7 | 8 |
|  |  | **Median (IQR)** | 0,2(0) | 0,2(0) | 0,15(0,1) | 0,6(0,4) | 0,4(0,4) | **0,3* (0,2)** |
|  | Normal | **n** | 6 | 6 | 3 | 35 | 34 | 32 |
|  |  | **Median (IQR)** | 0,3(0,2) | **0,2* (0,1)** | 0,2(0,2) | 0,4(0,3) | 0,4 (0,2) | **0,3*$ (0,1)** |
|  | Overweight | **n** | 2 | 2 | 2 | 19 | 17 | 17 |
|  |  | **Median (IQR)** | 0,3(0,5) | 0,2(0) | 0,3(0,2) | 0,4(0,4) | 0,3(0,2) | 0,3(0,2) |
| **Symptoms** | No | **n** | 3 | 2 | 2 | 11 | 10 | 6 |
|  |  | **Median (IQR)** | 0,2(0,1) | 0,25(0,1) | 0,2(0) | 0,4 (0,4) | 0,4 (0,3) | 0,3 (0,2) |
|  | Yes | **n** | 9 | 10 | 7 | 67 | 60 | 54 |
|  |  | **Median (IQR)** | 0,4(0,2) | 0,2 (0) | 0,2 (0,2) | 0,4(0,4) | 0,4 (0,2) | **0,3*$ (0,2)** |
| **Chest pain** | No | **n** | 10 | 10 | 7 | 52 | 45 | 38 |
|  |  | **Median (IQR)** | 0,2(0,2) | 0,2(0,1) | 0,2 (0) | 0,4(0,3) | 0,3 (0,2) | **0,3*$ (0,2)** |
|  | Yes | **n** | 2 | 2 | 2 | 23 | 22 | 20 |
|  |  | **Median (IQR)** | 0,5(0,2) | 0,2(0) | 0,2(0,3) | 0,5(0,5) | 0,4(0,2) | **0,3*$ (0,2)** |
| **Productive cough** | No | **n** | 8 | 8 | 7 | 38 | 35 | 30 |
|  |  | **Median (IQR)** | 0,35(0,2) | **0,2* (0,2)** | **0,2*(0,2)** | 0,4(0,4) | 0,4(0,1) | **0,3*$ (0,2)** |
|  | Yes | **n** | 4 | 4 | 2 | 39 | 34 | 29 |
|  |  | **Median (IQR)** | 0,2(0,25) | 0,2(0,2) | 0,2(0) | 0,5(0,5) | 0,4 (0,3) | **0,3*$ (0,2)** |
| **Haemoptysis** | No | **n** | 12 | 12 | 9 | 61 | 56 | 50 |
|  |  | **Median (IQR)** | 0,3(0,2) | 0,2 (0,15) | 0,2(0) | 0,4(0,3) | 0,4(0,2) | **0,3*$ (0,2)** |
|  | Yes | **n** | 0 | 0 | 0 | 12 | 11 | 7 |
|  |  | **Median (IQR)** | . (.) | . (.) | . (.) | 0,5(0,5) | 0,3(0,2) | **0,3*$ (0,2)** |
| **Night Sweats** | No | **n** | 11 | 11 | 8 | 50 | 44 | 35 |
|  |  | **Median (IQR)** | 0,3(0,2) | **0,2* (0)** | 0,2 (0,1) | 0,4(0,3) | 0,4(0,1) | **0,3*$ (0,2)** |
|  | Yes | **n** | 1 | 1 | 1 | 24 | 23 | 22 |
|  |  | **Median (IQR)** | 0,3(0) | 0,4(0) | 0,2(0) | 0,5(0,5) | **0,4* (0,3)** | **0,3*$ (0,2)** |
| **Shortness of breath** | No | **n** | 10 | 10 | 7 | 60 | 54 | 46 |
|  |  | **Median (IQR)** | 0,3(0,2) | 0,2 (0,1) | **0,2*$ (0,1)** | 0,4(0,3) | **0,4* (0,2)** | **0,3*$ (0,2)** |
|  | Yes | **n** | 2 | 2 | 2 | 16 | 14 | 13 |
|  |  | **Median (IQR)** | 0,3(0,5) | 0,2(0) | 0,3(0,2) | 0,5(0,4) | 0,4(0,3) | **0,3*$ (0,1)** |
| **Constitutional syndrome** | No | **n** | 5 | 5 | 4 | 38 | 34 | **26** |
|  |  | **Median (IQR)** | 0,2(0,1) | 0,2(0,1) | 0,2(0,05) | 0,3(0,2) | 0,3(0,1) | **0,3 $(0,2)** |
|  | Yes | **n** | 7 | 7 | 5 | 36 | 33 | 33 |
|  |  | **Median (IQR)** | 0,4(0,2) | 0,2(0) | **0,2* (0,1)** | 0,5(0,4) | **0,4* (0,3)** | **0,3*$ (0,2)** |
| **Feverish feeling** | No | **n** | 9 | 9 | 6 | 38 | 33 | 26 |
|  |  | **Median (IQR)** | 0,3(0,2) | 0,2(0,1) | 0,2 (0) | 0,4(0,4) | 0,4(0,2) | **0,3*$ (0,2)** |
|  | Yes | **n** | 3 | 3 | 3 | 38 | 35 | 33 |
|  |  | **Median (IQR)** | 0,4(0,4) | 0,2(0) | 0,1(0,3) | 0,5(0,4) | **0,4* (0,2)** | **0,3*$ (0,2)** |
| **Lymphadenopathy** | No | **n** | 10 | 9 | 6 | 65 | 58 | 51 |
|  |  | **Median (IQR)** | 0,3(0,2) | **0,2* (0)** | 0,2 (0) | 0,5(0,4) | **0,4* (0,2)** | **0,3*$ (0,2)** |
|  | Yes | **n** | 1 | 2 | 2 | 12 | 11 | 9 |
|  |  | **Median (IQR)** | 0,2(0) | 0,25(0,1) | 0,2(0,2) | 0,2(0,05) | 0,3(0,1) | 0,3(0,2) |

CH50

|  |  |  | **CHILDREN** | | | **ADULTS** | | |
| --- | --- | --- | --- | --- | --- | --- | --- | --- |
|  |  |  | **Baseline** | **FUM2** | **FUM6** | **Baseline** | **FUM2** | **FUM6** |
| **Sex** | Men | **n** | 4 | 8 | 4 | 25 | 21 | 22 |
|  |  | **Median (IQR)** | 69 (52,5) | 60,15 (23,19) | 54,22 (17,96) | 68,33 (17,77) | 74,8 (17,99) | **66,56$ (13,49)** |
|  | Women | **n** | 1 | 2 | 2 | 12 | 9 | 14 |
|  |  | **Median (IQR)** | 56 (0) | 54,47 (3,34) | 49,45 (5,1) | 68,28 (18,48) | 66,93 (9,01) | **63,8* (10,69)** |
| **Age** | <18 | **n** | 5 | 10 | 6 | 0 | 0 | 0 |
|  |  | **Median (IQR)** | 56 (34) | 55,22 (14,95) | 51,68 (8,29) | - (-) | - (-) | - (-) |
|  | 18 - 40 | **n** | 0 | 0 | 0 | 13 | 11 | 11 |
|  |  | **Median (IQR)** | . (.) | . (.) | . (.) | 73,38 (22,88) | 71,27 (31,7) | **58,87*$ (17,06)** |
|  | 41    – 60 | **n** | 0 | 0 | 0 | 20 | 14 | 22 |
|  |  | **Median (IQR)** | . (.) | . (.) | . (.) | 66,75 (18,49) | 73,3 (12,38) | **66,16$ (9,66)** |
|  | > 60 | **n** | 0 | 0 | 0 | 4 | 5 | 3 |
|  |  | **Median (IQR)** | . (.) | . (.) | . (.) | 69,95 (8,91) | 67,37 (11,61) | 64,01 (8,4) |
| **Smoking habit** | Never smoked | **n** | 0 | 0 | 0 | 18 | 24 | 22 |
|  |  | **Median (IQR)** | . (.) | . (.) | . (.) | 4 (20) | 64,61 (17,58) | 61,46 (12,78) |
|  | Ex-smoker | **n** | 0 | 0 | 0 | 4 | 2 | 3 |
|  |  | **Median (IQR)** | . (.) | . (.) | . (.) | 51,42 (17,13) | 61,99 (15,96) | 45,67 (7,99) |
|  | Active smoker | **n** | 0 | 0 | 0 | 20 | 14 | 17 |
|  |  | **Median (IQR)** | . (.) | . (.) | . (.) | 71,16 (16,68) | 76,36 (18,53) | **66,68*$ (12,33)** |
| **Alcohol intake** | No | **n** | 0 | 0 | 0 | 29 | 30 | 30 |
|  |  | **Median (IQR)** | . (.) | . (.) | . (.) | 68,23 (23,51) | 64,6 (16,89) | **59,42*$ (16,74)** |
|  | Yes | **n** | 0 | 0 | 0 | 13 | 10 | 12 |
|  |  | **Median (IQR)** | . (.) | . (.) | . (.) | 69,56 (15,46) | **76,39* (8,14)** | **67,86$ (8,11)** |
| **Country of origin** | Out of Spain | **n** | 1 | 2 | 2 | 22 | 16 | 20 |
|  |  | **Median (IQR)** | 86 (0) | 63,65 (18,7) | 68,05 (21,91) | 68,2 (15,83) | 67,14 (19,86) | **62,90*$ (15,68)** |
|  | Spain | **n** | 4 | 8 | 4 | 14 | 13 | 15 |
|  |  | **Median (IQR)** | 54 (37,5) | 54,47 (19,69) | 50,08 (3,83) | 68,28 (19,73) | 71,85 (10,06) | 66,43 (10,94) |
| **Comorbidities** | Yes | **n** | 0 | 1 | 0 | 13 | 10 | 10 |
|  |  | **Median (IQR)** | . (.) | 41,56 (0) | . (.) | 66,24 (9,6) | 69,2 (13,06) | 68,62 (9,14) |
|  | No | **n** | 3 | 5 | 3 | 23 | 17 | 26 |
|  |  | **Median (IQR)** | 56 (34) | 52,8 (2,01) | 51,36 (10,19) | 68,33 (17,77) | 71,27 (19,61) | **63,46*$ (11,25)** |
| **Previous exposure to TB drugs** | Yes | **n** | 0 | 0 | 0 | 1 | 0 | 0 |
|  |  | **Median (IQR)** | . (.) | . (.) | . (.) | 66,28 (0) | . (.) | . (.) |
|  | No | **n** | 5 | 10 | 6 | 36 | 30 | 36 |
|  |  | **Median (IQR)** | 56 (34) | 55,22 (14,95) | 51,68 (8,29) | 68,33 (18,85) | 70,88 (17,94) | **64,82*$ (12,68)** |
| **Tuberculin history** | Negative | **n** | 0 | 1 | 0 | 2 | 3 | 2 |
|  |  | **Median (IQR)** | . (.) | 41,56 (0) | . (.) | 63,98 (13,52) | 57,36 (17,56) | 54,06 (18,51) |
|  | Positive | **n** | 2 | 6 | 3 | 3 | 3 | 6 |
|  |  | **Median (IQR)** | 63,5 (71) | 66,62 (16,86) | 52 (30,2) | 88,19 (27,23) | 70,49 (32,14) | 66,87 (24,9) |
|  | Never done | **n** | 3 | 3 | 3 | 29 | 22 | 26 |
|  |  | **Median (IQR)** | 56 (34) | 52,8 (2,01) | 51,36 (10,19) | 68,23 (14,46) | 71,56 (12,28) | **63,81*$ (11,38)** |
| **Type of diagnosis** | Clinical | **n** | 2 | 6 | 3 | 7 | 6 | 9 |
|  |  | **Median (IQR)** | 77,5 (43) | 54,47 (31,44) | 52 (32,1) | 66,24 (19,02) | 65,07 (11,95) | **57,41 (13,23)** |
|  | Microbiological | **n** | 3 | 4 | 3 | 30 | 23 | 27 |
|  |  | **Median (IQR)** | 52 (58) | 60,15 (13,32) | 51,36 (8,29) | 68,95 (17,98) | 74,75 (25,53) | **66,43*$ (13,49)** |
| **Type of TB** | Pulmonary | **n** | 2 | 5 | 2 | 23 | 20 | 23 |
|  |  | **Median (IQR)** | 77,5 (43) | 56,14 (20,2) | 62,95 (32,1) | 68,32 (17,96) | 71,56 (19,78) | **64,01*$ (9,92)** |
|  | ExtraP + pulmonary | **n** | 1 | 1 | 2 | 1 | 1 | 2 |
|  |  | **Median (IQR)** | 52 (0) | 52,29 (0) | 51,68 (0.64) | 44,54 (0) | 69,97 (0) | 46,49 (14,21) |
|  | Extrapulmonary | **n** | 1 | 3 | 1 | 12 | 7 | 10 |
|  |  | **Median (IQR)** | 28 (0) | 66 (35,67) | 48,8 (0) | 69,92 (25,95) | 67,91 (15,05) | 68,28 (26,95) |
|  | Disseminated | **n** | 1 | 1 | 1 | 1 | 2 | 1 |
|  |  | **Median (IQR)** | 86 (0) | 54,03 (0) | 57,09 (0) | 69,56 (0) | 85,54 (25,58) | 68,9 (0) |
| **Culture** | Negative | **n** | 1 | 5 | 3 | 3 | 2 | 3 |
|  |  | **Median (IQR)** | 56 (0) | 52,8 (14,58) | 52 (32,1) | 66,28 (28,56) | 68,61 (9,2) | 55,67 (41,44) |
|  | Positive | **n** | 4 | 5 | 3 | 32 | 26 | 32 |
|  |  | **Median (IQR)** | 69 (52,5) | 66 (12,93) | 51,36 (8,29) | 68,33 (17,87) | 70,88 (17,94) | **66,03*$ (11,25)** |
| **TB-drug resistance** | Sensitive | **n** | 3 | 3 | 2 | 26 | 23 | 28 |
|  |  | **Median (IQR)** | 86 (47) | 54,3 (46,71) | 54,22 (5,73) | 68,33 (16,59) | 70,49 (20,95) | **64,82*$ (11,25)** |
|  | MDR or monoR | **n** | 1 | 2 | 1 | 4 | 2 | 3 |
|  |  | **Median (IQR)** | 28 (0) | 66,62 (1,23) | 48,8 (0) | 71,07 (24,72) | 78,88 (10,05) | 78,88 (29,22) |
| **AFB** | Negative | **n** | 4 | 9 | 6 | 13 | 12 | 15 |
|  |  | **Median (IQR)** | 54 (31) | 54,3 (13,71) | 51,68 (8,29) | 67,04 (14,7) | 65,56 (18,84) | 63,61 (12,78) |
|  | Positive | **n** | 1 | 1 | 0 | 22 | 14 | 20 |
|  |  | **Median (IQR)** | 99 (0) | 99 (0) | . (.) | 68,28 (18,55) | 71,56 (13,06) | **66,03*$ (11,95)** |
| **BMI** | Underweigth | **n** | 1 | 1 | 1 | 4 | 2 | 4 |
|  |  | **Median (IQR)** | 28 (0) | 67,23 (0) | 48,8 (0) | 76,43 (30,18) | 72,87 (31,02) | 59,75 (43,33) |
|  | Normal | **n** | 1 | 5 | 2 | 25 | 21 | 22 |
|  |  | **Median (IQR)** | 52 (0) | 52,29 (14,58) | 51,68 (0,64) | 69,56 (15,67) | 71,85 (11,94) | **66,78*$ (12,48)** |
|  | Overweight | **n** | 1 | 2 | 2 | 7 | 5 | 10 |
|  |  | **Median (IQR)** | 56 (0) | 62,9 (20,2) | 62,95 (32,1) | 60,23 (9,06) | 57,24 (2,46) | 62,76 (12,04) |
| **Symptoms** | No | **n** | 0 | 0 | 0 | 4 | 6 | 4 |
|  |  | **Median (IQR)** | . (.) | . (.) | . (.) | 68,3 (21,13) | 74,78 (8) | 66,26 (12,85) |
|  | Yes | **n** | 5 | 10 | 6 | 33 | 24 | 32 |
|  |  | **Median (IQR)** | 56 (34) | 55,22 (14,95) | 51,68 (8,29) | 68,32 (18,55) | 69,2 (19,78) | **64,82*$ (13,71)** |
| **Chest pain** | No | **n** | 4 | 8 | 4 | 25 | 19 | 24 |
|  |  | **Median (IQR)** | 54 (31) | 53,55 (14,15) | 50,08 (6,37) | 68,32 (15,83) | 74,75 (21,63) | **66,16*$ (12,48)** |
|  | Yes | **n** | 0 | 1 | 2 | 12 | 11 | 12 |
|  |  | **Median (IQR)** | . (.) | 73 (0) | 65,5 (27) | 69,12 (23,39) | 67,91 (10,92) | 68,5 (16,3) |
| **Productive cough** | No | **n** | 3 | 6 | 5 | 14 | 12 | 16 |
|  |  | **Median (IQR)** | 52 (58) | 60,15 (14,95) | 52 (5,73) | 66,75 (27,96) | 72,62 (21,1) | 65,02 (16,22) |
|  | Yes | **n** | 2 | 4 | 1 | 23 | 18 | 20 |
|  |  | **Median (IQR)** | 77,5 (43) | 54,47 (35,57) | 46,9 (0) | 68,33 (16,59) | 69,32 (17,94) | **64,82*$ (11,89)** |
| **Haemoptysis** | No | **n** | 5 | 10 | 6 | 31 | 22 | 29 |
|  |  | **Median (IQR)** | 56 (34) | 55,22 (14,95) | 51,68 (8,29) | 68,23 (15,29) | 71,17 (14,76) | **64,01*$ (16,04)** |
|  | Yes | **n** | 0 | 0 | 0 | 5 | 7 | 6 |
|  |  | **Median (IQR)** | . (.) | . (.) | . (.) | 80,73 (17,77) | 71,27 (27,69) | **66,66$ (7,32)** |
| **Night Sweats** | No | **n** | 4 | 9 | 6 | 25 | 19 | 22 |
|  |  | **Median (IQR)** | 54 (31) | 54,3 (17,71) | 51,68 (8,29) | 67,04 (18,37) | 71,85 (13,06) | **66,56*$ (10,03)** |
|  | Yes | **n** | 1 | 1 | 0 | 11 | 10 | 13 |
|  |  | **Median (IQR)** | 99 (0) | 99 (0) | . (.) | 70,74 (23,94) | 64,36 (14,93) | 64,01 (17,28) |
| **Shortness of breath** | No | **n** | 3 | 7 | 4 | 26 | 22 | 27 |
|  |  | **Median (IQR)** | 52 (58) | 54,3 (24,44) | 51,68 (4,46) | 67,55 (16,42) | 70,23 (20,11) | 66,43 (12,84) |
|  | Yes | **n** | 1 | 2 | 2 | 11 | 8 | 9 |
|  |  | **Median (IQR)** | 56 (0) | 62,9 (20,2) | 62,95 (32,1) | 71,78 (16,31) | 73,96 (21,85) | **59,24*$ (13,33)** |
| **Constitutional syndrome** | No | **n** | 2 | 4 | 2 | 22 | 18 | 19 |
|  |  | **Median (IQR)** | 54 (4) | 54,47 (8,53) | 49,13 (4,46) | 68,94 (16,59) | 72,62 (18,62) | **66,43*$ (6,69)** |
|  | Yes | **n** | 3 | 6 | 4 | 14 | 11 | 16 |
|  |  | **Median (IQR)** | 86 (71) | 60,77 (31,44) | 54,55 (17,64) | 67,34 (25,11) | 71,27 (18,27) | **62,43$ (19,12)** |
| **Feverish feeling** | No | **n** | 4 | 8 | 3 | 17 | 14 | 15 |
|  |  | **Median (IQR)** | 71 (38,5) | 53,55 (14,15) | 51,36 (10,19) | 67,04 (19,46) | 73,3 (20,95) | 66,68 (5,48) |
|  | Yes | **n** | 1 | 2 | 3 | 19 | 15 | 21 |
|  |  | **Median (IQR)** | 28 (0) | 70,12 (5,77) | 52 (30,2) | 68,33 (16,52) | 67,37 (27,01) | **59,6*$ (18,87)** |
| **Lymphadenopathy** | No | **n** | 2 | 6 | 4 | 29 | 25 | 31 |
|  |  | **Median (IQR)** | 71 (30) | 53,55 (14,58) | 54,55 (18,6) | 68,33 (15,13) | 70,49 (16,1) | **63,61*$ (13,23)** |
|  | Yes | **n** | 2 | 3 | 2 | 7 | 4 | 5 |
|  |  | **Median (IQR)** | 40 (24) | 66 (14,95) | 50,08 (2,55) | 60,23 (24,24) | 64,55 (25,58) | 68,79 (18,66) |

C3

|  |  |  | **CHILDREN** | | | **ADULTS** | | |
| --- | --- | --- | --- | --- | --- | --- | --- | --- |
|  |  |  | **Baseline** | **FUM2** | **FUM6** | **Baseline** | **FUM2** | **FUM6** |
| **Sex** | Men | **n** | 9 | 10 | 7 | 35 | 33 | 25 |
|  |  | **Median (IQR)** | 124,1 (35) | 131,45 (39,1) | 119,4 (41,1) | 150 (64) | **148* (38,8)** | **134,09$ (38,78)** |
|  | Women | **n** | 1 | 2 | 2 | 18 | 15 | 15 |
|  |  | **Median (IQR)** | 144 (0) | 121 (4) | 104,25 (37,5) | 160,5 (55) | **134* (47,67)** | **125*$ (24,94)** |
| **Age** | <18 | **n** | 10 | 12 | 9 | 0 | 0 | 0 |
|  |  | **Median (IQR)** | 125,05 (36) | 126,45 (28,55) | **119,4* (25)** | - (-) | - (-) | - (-) |
|  | 18 - 40 | **n** | 0 | 0 | 0 | 18 | 19 | 13 |
|  |  | **Median (IQR)** | . (.) | . (.) | . (.) | 153 (51) | **130* (39,17)** | **127*$ (44,78)** |
|  | 41    – 60 | **n** | 0 | 0 | 0 | 29 | 23 | 24 |
|  |  | **Median (IQR)** | . (.) | . (.) | . (.) | 160 (26) | **145* (39,8)** | **130,55*$ (27,5)** |
|  | > 60 | **n** | 0 | 0 | 0 | 6 | 6 | 3 |
|  |  | **Median (IQR)** | . (.) | . (.) | . (.) | 156,5 (101) | 161,43 (102,2) | 127 (52,34) |
| **Smoking habit** | Never smoked | **n** | 0 | 0 | 0 | 33 | 32 | 26 |
|  |  | **Median (IQR)** | . (.) | . (.) | . (.) | 143 (53) | **133,5* (36,8)** | **122,02*$ (29,9)** |
|  | Ex-smoker | **n** | 0 | 0 | 0 | 4 | 5 | 4 |
|  |  | **Median (IQR)** | . (.) | . (.) | . (.) | 158 (28) | 122 (15) | 122 (15,14) |
|  | Active smoker | **n** | 0 | 0 | 0 | 26 | 23 | 19 |
|  |  | **Median (IQR)** | . (.) | . (.) | . (.) | 153 (56) | **148* (43,87)** | **135*$ (42,65)** |
| **Alcohol intake** | No | **n** | 0 | 0 | 0 | 44 | 40 | 35 |
|  |  | **Median (IQR)** | . (.) | . (.) | . (.) | 148,5 (56,45) | **133* (37,3)** | **127*$ (37)** |
|  | Yes | **n** | 0 | 0 | 0 | 18 | 19 | 14 |
|  |  | **Median (IQR)** | . (.) | . (.) | . (.) | 150 (46) | **148* (39,8)** | **128,5*$ (35)** |
| **Country of origin** | Out of Spain | **n** | 2 | 2 | 2 | 29 | 26 | 24 |
|  |  | **Median (IQR)** | 151 (50) | 145,3 (16,6) | 123,2 (7,6) | 161 (41) | **148* (40)** | **132,54*$ (41,39)** |
|  | Spain | **n** | 8 | 10 | 7 | 23 | 21 | 15 |
|  |  | **Median (IQR)** | 120,55 (44,3) | 122,86 (25) | 103,3 (51,9) | 148 (81) | **140* (42)** | **125*$ (35)** |
| **Comorbidities** | Yes | **n** | 1 | 1 | 0 | 18 | 13 | 12 |
|  |  | **Median (IQR)** | 90,4 (0) | 83,5 (0) | . (.) | 150 (67) | 164 (44,47) | **134.55* (32,39)** |
|  | No | **n** | 7 | 7 | 5 | 29 | 29 | 27 |
|  |  | **Median (IQR)** | 126 (36) | 123 (29) | 123 (25) | 156 (40) | **145* (27,21)** | **126*$ (37)** |
| **Previous exposure to TB drugs** | Yes | **n** | 0 | 0 | 0 | 4 | 3 | 0 |
|  |  | **Median (IQR)** | . (.) | . (.) | . (.) | 120 (60,5) | 129 (122) | . (.) |
|  | No | **n** | 10 | 12 | 9 | 49 | 44 | 40 |
|  |  | **Median (IQR)** | 125,05 (36) | 126,45 (28,55) | **119,4* (25)** | 160 (55) | **148* (41)** | **128,55*$ (33,48)** |
| **Tuberculin history** | Negative | **n** | 2 | 1 | 0 | 2 | 3 | 2 |
|  |  | **Median (IQR)** | 108,2 (35,6) | 83,5 (0) | . (.) | 133,5 (45) | 121 (50,2) | 107,52 (0,96) |
|  | Positive | **n** | 5 | 8 | 6 | 9 | 8 | 5 |
|  |  | **Median (IQR)** | 117 (16,1) | 126,45 (34,99) | 102,65 (28,2) | 149 (34) | 144,11 (51,26) | **130,1* (47)** |
|  | Never done | **n** | 3 | 3 | 3 | 38 | 33 | 31 |
|  |  | **Median (IQR)** | 144 (33) | **133* (18)** | 127 (24,02) | 163,5 (53) | 148 (34,22) | **127*$ (27)** |
| **Type of diagnosis** | Clinical | **n** | 7 | 8 | 5 | 10 | 9 | 9 |
|  |  | **Median (IQR)** | 117 (53,6) | 121 (43,85) | 102 (28,2) | 172 (51) | **134* (38,22)** | **125* (15)** |
|  | Microbiological | **n** | 3 | 4 | 4 | 43 | 38 | 31 |
|  |  | **Median (IQR)** | 143 (51,9) | 131,45 (6,69) | 135,05 (29,92) | 150 (57) | **148,5* (43)** | **130,1*$ (46,78)** |
| **Type of TB** | Pulmonary | **n** | 6 | 32 | 7 | 31 | 4 | 25 |
|  |  | **Median (IQR)** | 121,5 (36) | 149,5 (54,5) | 123 (45,6) | 145 (42) | **110,7* (24,6)** | **127*$ (33)** |
|  | ExtraP + pulmonary | **n** | 1 | 3 | 1 | 3 | 2 | 3 |
|  |  | **Median (IQR)** | 143 (0) | 197 (49) | 133 (0) | 148 (110,52) | 116,26 (61,51) | 119 (79) |
|  | Extrapulmonary | **n** | 2 | 15 | 3 | 11 | 2 | 11 |
|  |  | **Median (IQR)** | 107,25 (33,7) | 150 (45) | 122,71 (46,4) | 130 (41,17) | **123,2* (39,8)** | **134,28* (47)** |
|  | Disseminated | **n** | 1 | 3 | 1 | 3 | 1 | 1 |
|  |  | **Median (IQR)** | 176 (0) | 210 (15) | 137 (0) | 190 (91) | 127 (0) | 148 (0) |
| **Culture** | Negative | **n** | 6 | 7 | 5 | 4 | 3 | 3 |
|  |  | **Median (IQR)** | 112,5 (35,6) | 119 (48,6) | 102 (28,2) | 165,5 (70) | 134 (67,76) | 134,28 (36) |
|  | Positive | **n** | 4 | 5 | 4 | 46 | 42 | 36 |
|  |  | **Median (IQR)** | 149,15 (32,1) | 133 (7,1) | 135,05 (29,92) | 153 (57) | **148* (44)** | **128,55*$ (36,5)** |
| **TB-drug resistance** | Sensitive | **n** | 3 | 3 | 2 | 38 | 37 | 31 |
|  |  | **Median (IQR)** | 155,3 (33) | 137 (14,1) | 137,01 (20,01) | 153 (57) | **145* (43)** | **130,1*$ (36)** |
|  | MDR or monoR | **n** | 1 | 2 | 2 | 6 | 3 | 4 |
|  |  | **Median (IQR)** | 124,1 (0) | 126,31 (7,18) | 123,2 (39,08) | 147,5 (79) | 165 (57,72) | 127,55 (59,05) |
| **AFB** | Negative | **n** | 9 | 11 | 9 | 22 | 18 | 15 |
|  |  | **Median (IQR)** | 124,1 (35) | 123 (29) | **119,4* (25)** | 145,5 (50) | 131 (28) | **130,1*$ (24)** |
|  | Positive | **n** | 1 | 1 | 0 | 28 | 25 | 24 |
|  |  | **Median (IQR)** | 155,3 (0) | 147,1 (0) | . (.) | 166,5 (64,5) | **152* (45,87)** | **124,02*$ (82,93)** |
| **BMI** | Underweigth | **n** | 2 | 5 | 2 | 4 | 2 | 5 |
|  |  | **Median (IQR)** | 116,05 (16,1) | 156 (47) | 115,36 (14,72) | 127 (60,12) | 102,65 (1,3) | 119 (27) |
|  | Normal | **n** | 5 | 29 | 6 | 29 | 4 | 24 |
|  |  | **Median (IQR)** | 117 (35,6) | 150 (46) | 126,45 (28) | 149 (41,8) | **117,15* (56,71)** | **133,5*$ (33,87)** |
|  | Overweight | **n** | 1 | 13 | 2 | 9 | 2 | 11 |
|  |  | **Median (IQR)** | 144 (0) | 149 (56) | 136,3 (34,6) | 134 (39) | 121,2 (3,6) | **127*$ (22,28)** |
| **Symptoms** | No | **n** | 3 | 2 | 2 | 6 | 7 | 4 |
|  |  | **Median (IQR)** | 117 (18) | 151 (86) | 96,6 (10,8) | 158,5 (78) | 158 (15) | 133,52 (14,42) |
|  | Yes | **n** | 7 | 10 | 7 | 47 | 41 | 36 |
|  |  | **Median (IQR)** | 143 (64,9) | 126,45 (18) | 123 (39,8) | 156 (61) | **136* (43)** | **127*$ (40,89)** |
| **Chest pain** | No | **n** | 9 | 10 | 7 | 35 | 31 | 27 |
|  |  | **Median (IQR)** | 124,1 (35) | 122,86 (25) | **123* (41,1)** | 150 (46) | **148* (33,17)** | **131*$ (40)** |
|  | Yes | **n** | 0 | 1 | 2 | 17 | 15 | 12 |
|  |  | **Median (IQR)** | . (.) | 153,6 (0) | 102,45 (33,9) | 179 (72) | **130* (45)** | **121,02* (24,64)** |
| **Productive cough** | No | **n** | 7 | 8 | 8 | 22 | 17 | 16 |
|  |  | **Median (IQR)** | 124,1 (35) | 131,45 (29,94) | 111,35 (38,45) | 148,5 (45) | **149* (38,17)** | **132,1* (34$)** |
|  | Yes | **n** | 3 | 4 | 1 | 30 | 30 | 23 |
|  |  | **Median (IQR)** | 144 (69,4) | 121 (23,05) | 123 (0) | 158 (64) | **138* (38,22)** | **127*$ (37)** |
| **Haemoptysis** | No | **n** | 10 | 12 | 9 | 40 | 38 | 32 |
|  |  | **Median (IQR)** | 125,05 (36) | 126,45 (28,55) | **119,4* (25)** | 150 (54,5) | **146,5* (43)** | **128,55*$ (32,98)** |
|  | Yes | **n** | 0 | 0 | 0 | 8 | 8 | 6 |
|  |  | **Median (IQR)** | . (.) | . (.) | . (.) | 133,5 (53) | 139 (34) | 131,5 (35,73) |
| **Night Sweats** | No | **n** | 9 | 11 | 9 | 32 | 28 | 22 |
|  |  | **Median (IQR)** | 124,1 (35) | 123 (29) | **119,4* (25)** | 148,5 (48) | **148,5* (38,9)** | **130,55*$ (27)** |
|  | Yes | **n** | 1 | 1 | 0 | 18 | 18 | 16 |
|  |  | **Median (IQR)** | 155,3 (0) | 147,1 (0) | . (.) | 161 (64) | **133,5* (31,22)** | **119,52* (26,5)** |
| **Shortness of breath** | No | **n** | 8 | 9 | 7 | 39 | 35 | 30 |
|  |  | **Median (IQR)** | 120,55 (35,3) | 123 (25) | 103,3 (51,9) | 156 (61) | **145* (43)** | **130,55*$ (35)** |
|  | Yes | **n** | 1 | 2 | 2 | 13 | 11 | 9 |
|  |  | **Median (IQR)** | 144 (0) | 136,3 (34,6) | 121,2 (3,6) | 150 (52) | 149 (42,22) | **125*$ (17)** |
| **Constitutional syndrome** | No | **n** | 5 | 6 | 5 | 29 | 24 | 19 |
|  |  | **Median (IQR)** | 126 (26) | 126,45 (14) | 123 (41,1) | 144 (45) | **149* (42)** | **131*$ (26,96)** |
|  | Yes | **n** | 5 | 6 | 4 | 23 | 22 | 20 |
|  |  | **Median (IQR)** | 124,1 (64,9) | 129,86 (42,1) | 111,35 (28,8) | 166 (58) | **135* (41,8)** | **124*$ (40)** |
| **Feverish feeling** | No | **n** | 9 | 10 | 6 | 25 | 20 | 17 |
|  |  | **Median (IQR)** | 126 (36) | 126,45 (29) | 125 (41,1) | 149 (41) | 149 (34,09) | **127$ (17,94)** |
|  | Yes | **n** | 1 | 2 | 3 | 27 | 27 | 23 |
|  |  | **Median (IQR)** | 124,1 (0) | 138,16 (30,88) | 103,3 (33,9) | 162 (62) | **134* (48,67)** | **134,28$ (44,78)** |
| **Lymphadenopathy** | No | **n** | 7 | 8 | 6 | 43 | 42 | 34 |
|  |  | **Median (IQR)** | 117 (53,6) | 121 (38,8) | 110,7 (31,8) | 150 (58) | **146,5* (39)** | **127*$ (33)** |
|  | Yes | **n** | 2 | 3 | 3 | 9 | 5 | 6 |
|  |  | **Median (IQR)** | 133,55 (18,9) | 129,9 (10,28) | 143,1 (47,72) | 161 (62) | 152,22 (57,2) | **455,55* (37,7)** |

C4

|  |  |  | **CHILDREN** | | | **ADULTS** | | |
| --- | --- | --- | --- | --- | --- | --- | --- | --- |
|  |  |  | **Baseline** | **FUM2** | **FUM6** | **Baseline** | **FUM2** | **FUM6** |
| **Sex** | Men | **n** | 9 | 10 | 7 | 35 | 33 | 25 |
|  |  | **Median (IQR)** | 30,7 (23) | 30,45 (16,7) | **24,83* (11,43)** | 32,7 (16,4) | **31,6* (14,6)** | **29,64*$ (10,81)** |
|  | Women | **n** | 1 | 2 | 2 | 18 | 15 | 15 |
|  |  | **Median (IQR)** | 28,2 (0) | 23,5 (0) | 16,8 (0) | 30,75 (8,4) | **26,4* (6,9)** | **21*$ (11,08)** |
| **Age** | <18 | **n** | 10 | 12 | 9 | 0 | 0 | 0 |
|  |  | **Median (IQR)** | 29,45 (23) | 26,85 (14,54) | **24* (8,1)** | . (.) | . (.) | . (.) |
|  | 18 - 40 | **n** | 0 | 0 | 0 | 18 | 19 | 13 |
|  |  | **Median (IQR)** | . (.) | . (.) | . (.) | 32,95 (12,2) | **30* (13)** | **27,02* (10,7)** |
|  | 41    – 60 | **n** | 0 | 0 | 0 | 29 | 23 | 24 |
|  |  | **Median (IQR)** | . (.) | . (.) | . (.) | 32,7 (14,1) | 30 (17,2) | **25,77*$ (11,75)** |
|  | > 60 | **n** | 0 | 0 | 0 | 6 | 6 | 3 |
|  |  | **Median (IQR)** | . (.) | . (.) | . (.) | 31,7 (11,4) | **27,15* (7,4)** | 25,52 (14,91) |
| **Smoking habit** | Never smoked | **n** | 0 | 0 | 0 | 33 | 32 | 26 |
|  |  | **Median (IQR)** | . (.) | . (.) | . (.) | 31,5 (11) | **27,9* (11,04)** | **24,91*$ (9,58)** |
|  | Ex-smoker | **n** | 0 | 0 | 0 | 4 | 5 | 4 |
|  |  | **Median (IQR)** | . (.) | . (.) | . (.) | 26,9 (22,2) | 20,3 (15) | 18,36 (18,81) |
|  | Active smoker | **n** | 0 | 0 | 0 | 26 | 23 | 19 |
|  |  | **Median (IQR)** | . (.) | . (.) | . (.) | 33,85 (14,1) | 31 (17,57) | **30,7*$ (18,36)** |
| **Alcohol intake** | No | **n** | 0 | 0 | 0 | 44 | 40 | 35 |
|  |  | **Median (IQR)** | . (.) | . (.) | . (.) | 31,8 (14,4) | **28,68* (12,2)** | **25*$ (11,33)** |
|  | Yes | **n** | 0 | 0 | 0 | 18 | 19 | 14 |
|  |  | **Median (IQR)** | . (.) | . (.) | . (.) | 33,85 (16,4) | 32,57 (15,5) | **30,29*$ (11,9)** |
| **Country of origin** | Out of Spain | **n** | 2 | 2 | 2 | 29 | 26 | 24 |
|  |  | **Median (IQR)** | 34,05 (18,1) | 33,5 (3,4) | 26,05 (4,1) | 34,1 (13,9) | **30,5* (15,67)** | **26,67*$ (11,98)** |
|  | Spain | **n** | 8 | 10 | 7 | 23 | 21 | 15 |
|  |  | **Median (IQR)** | 29,45 (20,75) | 23,5 (14,89) | **23,7* (19,53)** | 32,7 (12,2) | **25,7* (15,4)** | **24,4*$ (11,73)** |
| **Comorbidities** | Yes | **n** | 1 | 1 | 0 | 18 | 13 | 12 |
|  |  | **Median (IQR)** | 14,5 (0) | 12,5 (0) | . (.) | 33,85 (12,9) | **30,4* (13,24)** | **32*$ (7,96)** |
|  | No | **n** | 7 | 7 | 5 | 29 | 29 | 27 |
|  |  | **Median (IQR)** | 28,2 (23) | 24,6 (12,39) | **23,4* (4)** | 30,5 (16) | **27,2* (11,6)** | **24,1*$ (10,21)** |
| **Previous exposure to TB drugs** | Yes | **n** | 0 | 0 | 0 | 4 | 3 | 0 |
|  |  | **Median (IQR)** | . (.) | . (.) | . (.) | 23 (13,7) | 28,6 (10) | . (.) |
|  | No | **n** | 10 | 12 | 9 | 49 | 44 | 40 |
|  |  | **Median (IQR)** | 29,45 (23) | 26,85 (14,54) | **24* (8,1)** | 32,9 (12,4) | **30* (14,75)** | **25,92*$ (12,5)** |
| **Tuberculin history** | Negative | **n** | 2 | 1 | 0 | 2 | 3 | 2 |
|  |  | **Median (IQR)** | 19,75 (10,5) | 12,5 (0) | . (.) | 31,8 (15,2) | 34,6 (14) | 27,16 (3,28) |
|  | Positive | **n** | 5 | 8 | 6 | 9 | 8 | 5 |
|  |  | **Median (IQR)** | 30,7 (18,3) | 26,85 (18,3) | 22,41 (16,2) | 35,2 (15) | **33,67* (16,45)** | 38 (7,35) |
|  | Never done | **n** | 3 | 3 | 3 | 38 | 33 | 31 |
|  |  | **Median (IQR)** | 38,1 (14,9) | 31,8 (10,99) | 24 (8,03) | 32,1 (9,6) | **28,76* (9,3)** | **24,4*$ (10,7)** |
| **Type of diagnosis** | Clinical | **n** | 7 | 8 | 5 | 10 | 9 | 9 |
|  |  | **Median (IQR)** | 25 (15,8) | 23,5 (14,85) | 20 (11,5) | 30,15 (15,5) | 24,13 (9,7) | **23,4* (4,23)** |
|  | Microbiological | **n** | 3 | 4 | 4 | 43 | **38** | 31 |
|  |  | **Median (IQR)** | 39,3 (5) | 35,59 (13,12) | 28,13 (11,2) | 32,9 (12,4) | **30,7* (13,04)** | **28,8* (13,31)** |
| **Type of TB** | Pulmonary | **n** | 6 | 7 | 4 | 32 | 31 | 25 |
|  |  | **Median (IQR)** | 26,6 (15,6) | 24,6 (14,2) | 21,7 (9,8) | 29,55 (9,9) | **27,2* (10,47)** | **23,59*$ (12,5)** |
|  | ExtraP + pulmonary | **n** | 1 | 1 | 2 | 3 | 3 | 3 |
|  |  | **Median (IQR)** | 38,1 (0) | 33,36 (0) | 20,82 (21,23) | 36 (23,6) | 30 (27,89) | 21,9 (30,06) |
|  | Extrapulmonary | **n** | 2 | 3 | 2 | 15 | 11 | 11 |
|  |  | **Median (IQR)** | 26,9 (24,8) | 18,8 (30,65) | 32,31 (14,97) | 34,4 (17,8) | **31* (18,7)** | **31* (12,48)** |
|  | Disseminated | **n** | 1 | 1 | 1 | 3 | 3 | 1 |
|  |  | **Median (IQR)** | 43,1 (0) | 31,8 (0) | 24 (0) | 42,4 (22,2) | 37 (14) | 30,7 (0) |
| **Culture** | Negative | **n** | 6 | 7 | 5 | 4 | 3 | 3 |
|  |  | **Median (IQR)** | 20,05 (13,3) | 22,4 (21,6) | 20 (11,5) | 33,25 (17,5) | **28,76* (28,07)** | 25,23 (23) |
|  | Positive | **n** | 4 | 5 | 4 | 46 | 42 | 36 |
|  |  | **Median (IQR)** | 38,7 (5,45) | 31,8 (4,29) | 28,13 (11,2) | 32,25 (12,4) | 29,8 (14,24) | **26,67*$ (12,5)** |
| **TB-drug resistance** | Sensitive | **n** | 3 | 3 | 2 | 38 | 37 | 31 |
|  |  | **Median (IQR)** | 38,1 (9,7) | 31,8 (4,29) | 27,72 (7,43) | 32,35 (12,2) | **28,6* (12,5)** | **27,02*$ (13,8)** |
|  | MDR or monoR | **n** | 1 | 2 | 2 | 6 | 3 | 4 |
|  |  | **Median (IQR)** | 39,3 (0) | 30,82 (24,65) | 32,31 (14,97) | 28,85 (21,3) | 39,4 (8,46) | 29,46 (15,84) |
| **AFB** | Negative | **n** | 9 | 11 | **9** | 22 | 18 | 15 |
|  |  | **Median (IQR)** | 28,2 (23) | 24,6 (16,7) | **24* (8,1)** | 32,1 (14,8) | 28,61 (20,2) | **31* (17,7)** |
|  | Positive | **n** | 1 | 1 | 0 | 28 | 25 | 24 |
|  |  | **Median (IQR)** | 33,4 (0) | 29,1 (0) | . (.) | 32,25 (11,9) | 29,6* (9,6) | **24,7*$ (8,84)** |
| **BMI** | Underweigth | **n** | 2 | 2 | 2 | 5 | 4 | 5 |
|  |  | **Median (IQR)** | 27,1 (24,4) | 28,37 (29,54) | 18,37 (12,93) | 31,8 (18) | 33,95 (12,37) | 28,8 (19,25) |
|  | Normal | **n** | 5 | 6 | 4 | 29 | 29 | 24 |
|  |  | **Median (IQR)** | 25 (15,6) | 22,8 (14,89) | 25,72 (20,52) | 33,7 (14,3) | **30* (14,6)** | **28,33*$ (11,29)** |
|  | Overweight | **n** | 1 | 2 | 2 | 13 | 9 | 11 |
|  |  | **Median (IQR)** | 28,2 (0) | 28,8 (12,8) | 25,75 (4,7) | 28,6 (10,6) | **25,7* (8,3)** | **25,23* (11,3)** |
| **Symptoms** | No | **n** | 3 | 2 | 2 | 6 | 7 | 4 |
|  |  | **Median (IQR)** | 25 (15,8) | 27,25 (27,3) | 15,95 (8,1) | 37,65 (17,4) | 37 (21,1) | 30,29 (9,6) |
|  | Yes | **n** | 7 | 10 | 7 | 47 | 41 | 36 |
|  |  | **Median (IQR)** | 33,4 (24,2) | 26,85 (12,38) | 24,83 (8,03) | 32,4 (12,2) | **28,76* (10,1)** | **25,37*$ (12,5)** |
| **Chest pain** | No | **n** | 9 | 10 | 7 | 35 | 31 | 27 |
|  |  | **Median (IQR)** | 28,2 (23) | 23,5 (14,89) | **24* (11,43)** | 30,5 (15,2) | **27,2* (12,87)** | **25*$ (13,5)** |
|  | Yes | **n** | 0 | 1 | 2 | 17 | 15 | 12 |
|  |  | **Median (IQR)** | . (.) | 35,2 (0) | 19,15 (17,9) | 34,8 (7,5) | **29,6* (18,4)** | **28,33* (10,35)** |
| **Productive cough** | No | **n** | 7 | 8 | 8 | 22 | 17 | 16 |
|  |  | **Median (IQR)** | 30,7 (24,4) | 32,59 (22) | **24,41* (13,82)** | 30,4 (17,9) | **35,57* (18,8)** | **30,29* (16,39)** |
|  | Yes | **n** | 3 | 4 | 1 | 30 | 30 | 23 |
|  |  | **Median (IQR)** | 28,2 (18,3) | 23,5 (5,15) | 23,4 (0) | 32,55 (9) | **28,68* (9,2)** | **24,4*$ (11,6)** |
| **Haemoptysis** | No | **n** | 10 | 12 | 9 | 40 | 38 | 32 |
|  |  | **Median (IQR)** | 29,45 (23) | 26,85 (14,54) | **24* (8,1)** | 33,3 (17,3) | **30* (14,24)** | **25,92*$ (13,3)** |
|  | Yes | **n** | 0 | 0 | 0 | 8 | 8 | 6 |
|  |  | **Median (IQR)** | . (.) | . (.) | . (.) | 29,75 (5,25) | 28,4 (8,07) | 26,01 (6,24) |
| **Night Sweats** | No | **n** | 9 | 11 | 9 | 32 | 28 | 22 |
|  |  | **Median (IQR)** | 28,2 (23) | 24,6 (16,7) | **24* (8,1)** | 32,25 (16,8) | **29,8* (15,53)** | **28,45*$ (11,89)** |
|  | Yes | **n** | 1 | 1 | 0 | 18 | 18 | 16 |
|  |  | **Median (IQR)** | 33,4 (0) | 29,1 (0) | . (.) | 33,6 (11,1) | **29,5* (10,5)** | **23,9* (11,84)** |
| **Shortness of breath** | No | **n** | 8 | 9 | 7 | 39 | 35 | 30 |
|  |  | **Median (IQR)** | 27,85 (23,7) | 24,6 (14,89) | **24* (19,53)** | 32,9 (15,9) | **28,6* (15,4)** | **27,56*$ (14,21)** |
|  | Yes | **n** | 1 | 2 | 2 | 13 | 11 | 9 |
|  |  | **Median (IQR)** | 28,2 (0) | 28,8 (12,8) | 25,75 (4,7) | 31,8 (5,5) | **29,6* (7,6)** | **23,59*$ (5,12)** |
| **Constitutional syndrome** | No | **n** | 5 | 6 | 5 | 29 | 24 | 19 |
|  |  | **Median (IQR)** | 28,2 (5,7) | 23,5 (14,89) | 23,4 (11,43) | 28,6 (14,8) | **30,2* (14,58)** | **27,02*$ (13,01)** |
|  | Yes | **n** | 5 | 6 | 4 | 23 | 22 | 20 |
|  |  | **Median (IQR)** | 33,4 (24,2) | 30,45 (14,2) | 24,41 (9,36) | 34,8 (14,1) | **28,53* (14,24)** | **25,11* (14,06)** |
| **Feverish feeling** | No | **n** | 9 | 10 | 6 | 25 | 20 | 17 |
|  |  | **Median (IQR)** | 28,2 (18,3) | 23,5 (13,3) | **23,7* (11,43)** | 32,7 (17,2) | 33,54 (16,53) | **29,88*$ (14,91)** |
|  | Yes | **n** | 1 | 2 | 3 | 27 | 27 | 23 |
|  |  | **Median (IQR)** | 39,3 (0) | 39,17 (7,95) | **24,83* (17,9)** | 32,4 (9,3) | **28,6* (10)** | **25* (11,9)** |
| **Lymphadenopathy** | No | **n** | 7 | 8 | 6 | 43 | 42 | 34 |
|  |  | **Median (IQR)** | 25 (15,8) | 23,5 (16,2) | 21,7 (12,1) | 32,4 (12,4) | **29,2* (12,87)** | **24,7*$ (13,5)** |
|  | Yes | **n** | 2 | 3 | 3 | 9 | 5 | 6 |
|  |  | **Median (IQR)** | 38,7 (1,2) | 33,39 (24,65) | 31,43 (14,97) | 32,9 (17,8) | **38,74* (13,43)** | 32 (16,5) |

Table S4: Differences in the SGRQ and Kessler-10 at BL between groups

|  |  | **SGRQ total score** | | | **Kesser-10 scale** | | |
| --- | --- | --- | --- | --- | --- | --- | --- |
|  |  | **N** | **Me** | **pvalue** | **N** | **Me** | **pvalue** |
| **Sex** | Men | 29 | 14,2 | 0,2 | 14 | 14 | 1 |
|  | Women | 9 | 46,2 |  | 46 | 15,5 |  |
| **Age** | 18-40 | 14 | 13,05 |  | 23 | 13 | - |
|  | 41-60 | 17 | 49 | **0,004** | 23 | 21 | **0,02** |
|  | >60 | 7 | 4,4 | 0,5 | 14 | 11 | 0,9 |
| **Country origen** | Spain | 18 | 43,65 | **0,06** | 25 | 20 | **0,04** |
|  | Out Spain | 19 | 12,3 |  | 34 | 12,5 |  |
| **Smoking** | Yes | 19 | 41 | 0,38 | 26 | 17 | 0,3 |
|  | No | 18 | 13,7 |  | 33 | 15 |  |
| **Alcohol intake** | Yes | 18 | 23,55 | 0,4 | 25 | 17 | 0,8 |
|  | No | 15 | 46,2 |  | 30 | 14 |  |
| **Comorbidities** | Yes | 7 | 6,2 | 0,36 | 17 | 10 | **0,04** |
|  | No | 31 | 25,4 |  | 43 | 17 |  |
| **Phyquiatric** | Yes | 4 | 33,3 | 0,3 | 6 | 28,5 | 0,08 |
|  | No | 34 | 23,55 |  | 54 | 14 |  |
| **C-reactive protein** | <5 mg/ml | 14 | 26,2 | 0,7 | 21 | 20 | 0,4 |
|  | >5 mg/ml | 19 | 25,4 |  | 30 | 15,5 |  |
| **ERS** | <20 mm/h | 4 | 10,6 | 0,2 | 4 | 16,5 | 0,7 |
|  | >20 mm/h | 22 | 38,45 |  | 32 | 18,5 |  |
| **AFB** | Positive | 21 | 41 | 0,3 | 31 | 17 | 0,9 |
|  | Negative | 16 | 12,75 |  | 23 | 14 |  |
| **Culture** | Positive | 37 | 21,7 | - | 57 | 15 | - |
|  | Negative | 0 | - |  | 0 | - |  |
| **Symptoms** | No | 6 | 5,25 | **0,02** | 8 | 13,5 | 0,8 |
|  | Yes | 32 | 40,95 |  | 51 | 16 |  |
| **Chest pain** | No | 28 | 20,9 | 0,5 | 40 | 14,5 | 0,8 |
|  | Yes | 8 | 47,65 |  | 17 | 20 |  |
| **Productive cough** | No | 16 | 10,6 | 0,1 | 22 | 12,5 | 0,6 |
|  | Yes | 22 | 46,25 |  | 36 | 16,5 |  |
| **Hemoptisis** | No | 29 | 20,1 | 0,3 | 42 | 14,5 | 0,4 |
|  | Yes | 8 | 46,7 |  | 13 | 20 |  |
| **Night sweets** | No | 23 | 21,7 | 0,7 | 37 | 14 | 0,4 |
|  | Yes | 15 | 40,9 |  | 19 | 17 |  |
| **Shortness breath** | No | 30 | 17,15 | **0,01** | 44 | 13,5 | 0,5 |
|  | Yes | 7 | 53 |  | 13 | 23 |  |
| **Constitutional syndrome** | No | 20 | 17,15 | 0,16 | 27 | 15 | 0,8 |
|  | Yes | 16 | 43,6 |  | 29 | 15 |  |
| **Feverishing feeling** | No | 21 | 20,1 | 0,7 | 27 | 19 | 0,4 |
|  | Yes | 17 | 25,4 |  | 31 | 14 |  |
| * p-value<0,05 when compared between groups (statistical test: Mann-Whitney test) | | | | | | | |

Table S5a: Cut-off of the analyzed parameters for predicting culture conversion at FUM2

|  | Cut off | Sensitivity | Specificity | AUC |
| --- | --- | --- | --- | --- |
| PCR | >= 0,82 | 85 | 22,2 | 0,7167 |
| ESR | >= 14 | 100 | 11,11 | 0,4644 |
| NLR | >=5,9 | 22,73 | 88,89 | 0,4797 |
| MLR | >=1,1 | 13 | 100 | 0,6020 |
| C3 | >=155,3 | 65 | 50 | 0,4984 |
| C4 | >=28,6 | 80 | 33,33 | 0,4884 |
| CH50 | >=68,06 | 70,95 | 50 | 0,4550 |
| Clinical severity | >=4 | 68,75 | 77,78 | 0,5991 |
| Kessler | >=15 | 50 | 42,86 | 0,3520 |
| SGRQ total score | >=40,9 | 53,85 | 71,43 | 0,4740 |

Table S5b: Cut-off of the analyzed parameters for predicting presence of symptoms at FUM2

|  | Cut off | Sensitivity | Specificity | AUC |
| --- | --- | --- | --- | --- |
| PCR | >=8,76 | 59,09 | 60,53 | 0,4496 |
| ESR | >=47 | 88,24 | 40 | 0,6154 |
| NLR | >=3,2 | 55,33 | 48,78 | 0,5069 |
| MLR | >=0,9 | 21,74 | 90,48 | 0,4379 |
| C3 | >=206 | 43,75 | 89,66 | 0,6045 |
| C4 | >=32,7 | 75 | 62,07 | 0,6437 |
| CH50 | >=71,78 | 58,33 | 75 | 0,5598 |
| Clinical severity | >=4 | 57,89 | 53,85 | 0,5300 |
| Kessler | >=12 | 81,25 | 42,31 | 0,5198 |

-Supplementary material 6a: **ROC curves for discriminating cases with culture conversion at FUM2**

- RCP

- ESR

- NLR

- MLR

- C3

- C4

- CH50

- Clinical severity

- Kessler-10

- SGRQ

- Supplementary material 6b: **ROC curves for discriminating cases with symptoms at FUM2**

- RCP

- ESR

- NLR

- MLR

- C3

- C4

- CH50

- Clinical severity

- Kessler-10

- Total score SGRQ
